# Development of a screening platform to discover natural products active against SARS-CoV-2 infection using lung organoid models

**DOI:** 10.1186/s40824-023-00357-y

**Published:** 2023-03-01

**Authors:** Joo-Eun Lee, Se Yun Jeong, Zijun Li, Hyun-Yi Kim, Hyun-Woo Kim, Min Jeong Yoo, Hee Joo Jang, Do-Kyun Kim, Namki Cho, Hee Min Yoo, Ki Hyun Kim

**Affiliations:** 1 College of Pharmacy, Chonnam National University, Gwangju, 61186 Republic of Korea; 2 School of Pharmacy, Sungkyunkwan University, Suwon, 16419 Republic of Korea; 3 Biometrology Group, Korea Research Institute of Standards and Science (KRISS), Daejeon, 34113 Republic of Korea; 4 NGeneS Inc., Ansan, 15495 Republic of Korea; 5 Korea Zoonosis Research Institute, Jeonbuk National University, Iksan, 54531 Republic of Korea; 6 Department of Precision Measurement, University of Science and Technology (UST), Daejeon, 34113 Republic of Korea

**Keywords:** Natural products, Screening platform, SARS-CoV-2, Antiviral activity, Lung organoid models

## Abstract

**Background:**

Natural products can serve as one of the alternatives, exhibiting high potential for the treatment and prevention of COVID-19, caused by SARS-CoV-2. Herein, we report a screening platform to test the antiviral efficacy of a natural product library against SARS-CoV-2 and verify their activity using lung organoids.

**Methods:**

Since SARS-CoV-2 is classified as a risk group 3 pathogen, the drug screening assay must be performed in a biosafety level 3 (BSL-3) laboratory. To circumvent this limitation, pseudotyped viruses (PVs) have been developed as replacements for the live SARS-CoV-2. We developed PVs containing spikes from Delta and Omicron variants of SARS-CoV-2 and improved the infection in an angiotensin-converting enzyme 2 (ACE2)-dependent manner. Human induced pluripotent stem cells (hiPSCs) derived lung organoids were generated to test the SARS-CoV-2 therapeutic efficacy of natural products.

**Results:**

Flavonoids from our natural product library had strong antiviral activity against the Delta- or Omicron-spike-containing PVs without affecting cell viability. We aimed to develop strategies to discover the dual function of either inhibiting infection at the beginning of the infection cycle or reducing spike stability following SARS-CoV-2 infection. When lung cells are already infected with the virus, the active flavonoids induced the degradation of the spike protein and exerted anti-inflammatory effects. Further experiments confirmed that the active flavonoids had strong antiviral activity in lung organoid models.

**Conclusion:**

This screening platform will open new paths by providing a promising standard system for discovering novel drug leads against SARS-CoV-2 and help develop promising candidates for clinical investigation as potential therapeutics for COVID-19.

**Graphical Abstract:**

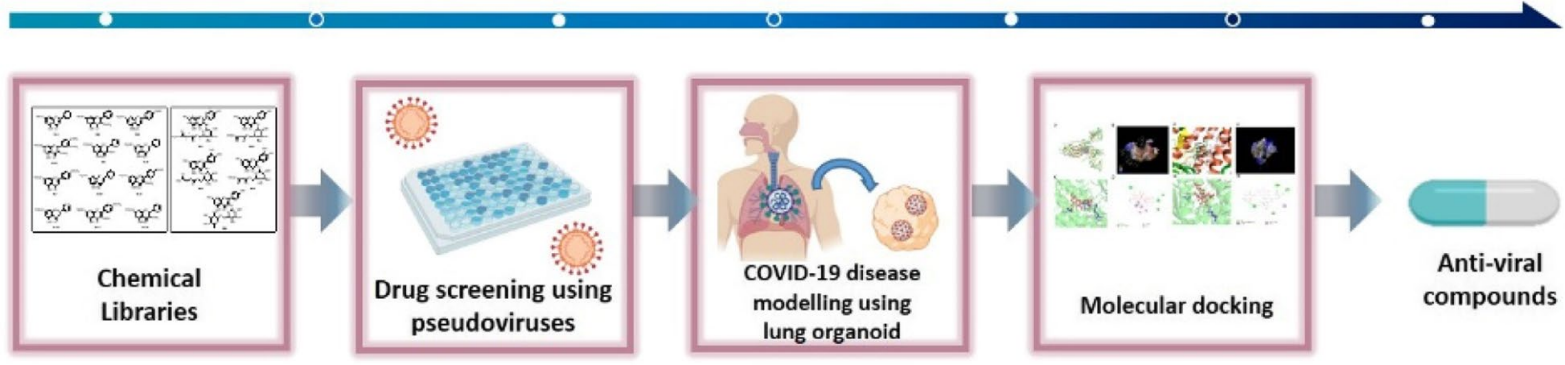

**Supplementary Information:**

The online version contains supplementary material available at 10.1186/s40824-023-00357-y.

## Introduction

Coronavirus disease 2019 (COVID-19), caused by severe acute respiratory syndrome coronavirus 2 (SARS-CoV-2), has spread globally, and new variants of the virus have been identified over the course of the pandemic [1]. SARS-CoV-2 is a positive-sense single-stranded RNA enveloped virus, and the genomic RNA is translated to produce structural and nonstructural proteins [2]. The S, E, M, and N genes encode structural proteins, and two open reading frames (ORFs), ORF1a and ORF1b, encode nonstructural proteins (nsps) [3]. The spike (S) protein of SARS-CoV-2, which mediates receptor binding and cell membrane fusion, is composed of two subunits, S1 and S2. The S1 subunit contains a receptor-binding domain (RBD) that recognizes and binds to angiotensin-converting enzyme 2 (ACE2) host receptor, while the S2 subunit facilitates fusion between the viral and host cell membranes [4, 5].

In principle, a neutralization assay is an effective technique for directly determining the inhibitory effect of antibodies or small molecules on viruses. In the case of SARS-CoV-2, neutralizing antibodies (nAbs) or small-molecule inhibitors primarily function by binding to the RBD within the spike protein, preventing the virus from attaching to the ACE2 receptor and gaining entry into the host cell [6]. Because SARS-CoV-2 has high pathogenicity and infectivity, inhibition assays involving live viruses must be performed under a biosafety level 3 (BSL-3) laboratory [7]. To circumvent this limitation, pseudotyped viruses (PVs) based on either a vesicular stomatitis virus (VSV) or lentiviral vector have been developed as a replacement for live SARS-CoV-2 [8]. The PVs are replication-defective viral particles that are made of an envelope and a part of the viral genome. Pseudotyping is accomplished by including the SARS-CoV-2 spike protein in the viral vector package, which can be assembled into the viral envelope of the PV, endowing it with an infection mechanism similar to SARS-CoV-2 [9].

The primary target tissues of SARS-CoV-2 appear to be lung alveoli in patients who develop pneumonia [10, 11]. The respiratory epithelium of tracheobronchial is pseudostratified and consists of specialized cells, including columnar club, cilia, goblet, and basal cells. Club cells (formerly known as Clara cells) are nonciliated and nonmucous cells, with short microvilli that produce immunomodulatory and anti-inflammatory proteins. Ciliated cells are lined across the apical surface and facilitate the movement of mucus across the respiratory tract. Goblet cells are mucin-producing cells that trap pathogens or particulates in the airway tract. Basal cells are progenitor cells that differentiate into other cell types to restore a healthy epithelial cell layer and respond to airway injury [12]. Organoids have proven extremely useful in modeling human diseases, studying host-pathogen interactions, and drug screening [13]. Particularly, lung organoids infected with SARS-CoV-2 can be used to study infection and provide a valuable resource for discovering COVID-19 therapeutics [14, 15]. In addition, human lung alveolar A549 cells overexpressing ACE2 are considered a classic and important model for respiratory virus infection [16]. To screen compounds with anti-infection activity, we used the A549-ACE2 cell line for initial screening and functional assays. The antiviral effects were confirmed using a lung organoid model.

Natural products have historically played a significant role in drug discovery, especially for cancer and infectious diseases [17, 18]. Natural products are structurally ‘optimized’ via evolution to serve particular biological functions, including the regulation of endogenous defense mechanisms and the interaction or competition with other organisms, which bestow them high potential for treatment and prevention of infectious diseases. Furthermore, their use in traditional medicine may provide insights into their efficacy and safety. As the COVID-19 pandemic continues, vaccines against COVID-19 are currently being developed and deployed [19], and the recently FDA-approved, direct-acting small-molecule antiviral drugs for SARS-CoV-2 including remdesivir, molnupiravir, and combination therapy using PF-07321332 with ritonavir (Paxlovid) are reportedly promising to reduce the risk of hospitalization and death [20–23]. However, considering the severe pathophysiology provoked by SARS-CoV-2 and the continuous emergence of its variants, the need for therapeutic alternatives that complement vaccination and FDA-approved drugs to alleviate and stop COVID-19 are required to control virus transmission and treat patients, which has shifted attention to natural products as novel drug candidates [24]. All coronavirus enzymes and proteins involved in viral replication and the regulation of host machinery are theoretically potential druggable targets for SARS-CoV-2 infection. Proteases such as 3-chymotrypsin-like protease (3CLpro) or papain-like protease (PLpro), spike (S) glycoprotein, RNA-dependent RNA polymerase (RdRp), and nonstructural proteins (nsps) have been identified as the druggable targets crucial for developing effective coronavirus therapeutics [24]. Among these targets, spike glycoprotein is crucial for binding and entry of the virus into the host and plays a pivotal role in SARS-CoV-2 host tropism and pathogenesis [25].

Recent reports have demonstrated potent in vitro anti-SARS-CoV-2 activity of diverse natural products such as terpenoids, polyketides, binaphthoquinones, peptides, and polyphenol-type structural classes [26–30]. Considered together, the molecular diversity represented by natural products hints at their potential for being a source of novel drugs, and they may satisfy the critical requirements of an ideal SARS-CoV-2 inhibitor. Although natural products are good candidates for antiviral efficacy, there is currently no effective screening platform to evaluate and verify their inhibitory efficacy against SARS-CoV-2 infection. In this study, we developed a screening platform to test the antiviral efficacy of various natural products against SARS-CoV-2 and verified it using lung organoids.

### Experimental section

#### General experimental procedures

See online Supplementary Information.

### Sample materials of *Kaempferia parviflora* rhizomes and *Morus alba* fruits

See online Supplementary Information*.*

### Extraction and isolation of natural products from *K. parviflora* rhizomes and *M. alba* fruits

See online Supplementary Information*.*

### Cell culture

The culture medium of HEK293T, BEAS-2B, and A549-ACE2 cells was Dulbecco’s modified Eagle’s medium (DMEM, Gibco) or Roswell Park Memorial Institute (RPMI, Gibco) 1640 medium supplemented with 10% fetal bovine serum (FBS, Gibco) and 1% (v/v) antibiotic antimycotic (Thermo Fisher Scientific, Waltham, MA, USA). All cells were incubated at 37 °C in a humidified atmosphere saturated with 5% CO_2_. Subcultures were generated when the cell density reached 70–80%, every three days.

### Microscopic analysis

Compounds-treated HEK293T, BEAS-2B, and A549-ACE2 cells were examined under the EVOS M5000 microscope (Thermo Fisher Scientific, Waltham, MA, USA) to detect morphological changes, as described in our previous study [31].

### Plasmids

The SARS-CoV-2-related sequences were codon-optimized for human expression and purchased from Sino Biological (Beijing, China). A GFP tag sequence was cloned into each of the synthesized genes to achieve high efficiency in detection. The SARS-CoV-2 spike coding sequences were cloned into the pMD2.G vector (Addgene, Watertown, MA, USA) to generate pMD2-S (containing the full-length spike sequence, depicted as S), pMD2-S-Delta (containing the full-length S with Delta variant, designated as S-Delta), and pMD2-S-Omicron (containing the full-length S with Omicron variant, designated as S-Omicron). The viral vector packaging plasmids psPAX2 and pMD2.G were obtained from Addgene.

### Pseudovirus production

Initially, pLVX-GFP, pLVX-S-Delta, or pLVX-S-Omicron was mixed with psPAX2 and VSV.G or spike variant-containing plasmid (pMD2-S-Delta or pMD2-S-Omicron) at a ratio of 4:3:1. The mixed plasmid DNA was then transfected into the cells using Metafectene PRO (Biontex, Munich, Germany). The viral supernatant was harvested 48 or 72 h after transfection. After cell debris was removed by filtration with a 0.45 μM filter, the supernatants containing the virus were stored at − 80 °C.

### Real-time quantitative polymerase chain reaction (RT-qPCR)

Total RNA was extracted from A549 cells 36 h after compound treatment using a Qiagen RNeasy mini kit (according to the manufacturer’s protocol). Equal amounts of RNA were used to synthesize cDNA using the LunaScript RT SuperMix Kit (New England Biolabs, Ipswich, MA, USA). NanoDrop spectrophotometer was used to measure the RNA concentration (Thermo Fisher Scientific, Waltham, MA, USA). The PCR was performed using specific primers and Ex Taq DNA polymerase reagent (Takara Bio, Kusatsu, Japan). To amplify the reaction, the StepOnePlus Real-Time PCR system (Thermo Fisher Scientific, Waltham, MA, USA) was used by following manufacturer’s instructions. Primer sequences used in this study (5′ → 3′) were IL-6 (F: AGCCCTGAGAAAGGAGACAT, R: TGGAAGGTTCAGGTTGTTTT), IL-1β (F: CTGTCCTGCGTGTTGAAAGA, R: TTCTGCTTGAGAGGTGCTGA), and TNF-α (F: AACCTCCTCTCTGCCATCAA, R: CCAAAGTAGACCTGCCCAGA). The F, R, and P indicate forward and reverse primers and probes, respectively.

### One-step real-time quantitative polymerase chain reaction (RT-qPCR)

Total RNA was extracted from A549 cells 36 h after compound treatment using a Qiagen RNeasy mini kit or QIAamp Viral RNA Kit (Qiagen, Hilden, Germany) according to the manufacturer’s protocol. RNA concentration was determined using a NanoDrop spectrophotometer (Thermo Fisher Scientific, Waltham, MA, USA). Specific primers, probes, and one-step RT-qPCR Master Mix (SFCprobes, Cheonju, Korea) were added to initiate the PCR. A StepOnePlus Real-Time PCR system (Thermo Fisher Scientific, Waltham, MA, USA) was used to amplify the reaction (according to the manufacturer’s instructions). The following primer sequences (5′ → 3′) were used in this study: Spike (F: TCTGCTTTACTAATGTCTATGC, R: GCTATAACGCAGCCTGTAAA, P: TCAGACAAATCGCTCCAGGGCA). The F, R, and P indicate forward and reverse primers and probes, respectively.

### Western blotting

The cells were then lysed and boiled for 15 min. Subsequently, the proteins were separated by sodium dodecyl sulfate-polyacrylamide gel electrophoresis (SDS-PAGE) and transferred to a PVDF membrane (0.45 μM). After blocking in 5% nonfat milk and washing with 1× TBST, the PVDF membrane was incubated with the primary antibodies (dilution ratio of 1:1000), purchased from Santa Cruz Biotechnology (Dallas, TX, USA), against GFP for spike-GFP fusion protein and actin. The ECL western blotting detection reagents (Immobilon Classico Western HRP substrate) were purchased from MilliporeSigma (Boston, MA, USA), and further analysis was performed using an ImageQuant software (Cytiva Korea, Seoul, Korea) and LAS 4000 mini imager (Fujifilm, Tokyo, Japan).

### Lung organoid (LO) culture

The hiPSC colonies were dissociated into single-cell suspensions using ReLeSR™. LOs were generated according to the modified manufacturer’s instructions using the commercial STEMdiff Branching Lung Organoid Kit (Stemcell Technologies, Vancouver, Canada). In brief, hiPSC differentiation in definitive endoderm was performed using Definitive Endoderm-1 (DE-1) and Definitive Endoderm-2 (DE-2) medium for 3 days. For anterior foregut endoderm (AFE) induction, the endoderm cells were cultured in Branching Lung Organoid-A (BLO-A) medium for 5–7 days. Free-floating AFE buds were then placed in individual wells of the suspension culture plate. The resulting AFE was incubated in Branching Lung Organoid-B (BLO-B) medium for days 6–14. For differentiation of mature lung cells, the day 15–36 culture was maintained at 37 °C with 5% CO_2_ and suspension conditions. Twice per week, a BLO-B medium change was performed. The morphology of LOs was monitored daily under a microscope.

### Confocal imaging

The cells treated with or without the compounds were fixed in 4% paraformaldehyde. The fixed cells were blocked with 5% bovine serum albumin (BSA) and permeabilized with 0.1% Triton X-100 in PBS. The primary antibodies against Oct3/4 (sc-5279), Nanog (sc-293,121), SOX-2, (sc-365,823), SOX-9 (sc-166,505), NKX2.1 (sc-53,136), Ep-CAM (sc-25,308), MUC5B (sc-21,768), Acetylated α-Tubulin (Ac tub, sc-23,950), CC10 (sc-365,992), SFTPB (sc-133,143), and caveolin-1 (sc-53,564) were purchased from Santa Cruz Biotechnology (USA). Cytokeratin 5 (Thermo Fisher Scientific, MA5–12596) was also purchased. Donkey anti-rabbit (or mouse) Alexa Fluor 488 and 594 (Jackson ImmunoResearch Laboratories, Inc. West Grove, PA, USA) were used as secondary antibodies. Both antibodies were used at a concentration of 1:100. Primary antibodies binding were performed overnight at 4 °C, and secondary anitbodies were diluted in blocking buffer and incuvate for 2 h in 25 °C. Subsequently, Hoechst 33342 (Thermo Fisher Scientific, Waltham, MA, USA) was used to stain the DNA. After mounting the samples coverslips were used to slides and sealed. Images were acquired using a confocal microscope (LSM800, Oberkochen, Germany) with Zen Blue Edition software (Zeiss, Oberkochen, Germany).

### Molecular docking

The structure of the Omicron S protein (PDB ID. 7TGW) was retrieved from the RCSB Protein Data Bank in PDB format. The 3D structure of 5-hydroxy-3,7,3′,4′-tetramethoxyflavone was constructed, and its energy minimization was performed via ChemOffice (PerkinElmer, Waltham, MA, USA). Both receptors and ligands were prepared using AutoDock4 and saved in the PDBQT format. As for 6M0J, the amino acids T478, E484, Q493, N501, G446, Q498, Y505, and N440 were chosen as active sites for the spike protein RBD because of their importance in escaping from neutralizing antibodies as per the previous report [32] and confined in the grid box of size 80 Å × 80 Å × 80 Å. Binding energies were optimized using the search genetic algorithm and Lamarckian genetic algorithm via auto-docking tools (AutoDock, GPL), and the results of molecular docking were visualized using PyMOL (Schrödinger, New York, NY, USA) and Discovery Studio software (BIOVIA, San Diego, CA, USA).

### Statistical analysis

Statistical analysis was performed using GraphPad Prism (GraphPad Software, Inc., San Diego, CA, USA.), and the values are presented as the mean ± SEM. The data were further analyzed using Student’s t-test. The *p*-values * *p* < 0.05, ** *p* < 0.01, *** *p* < 0.001, **** *p* < 0.0001 were considered statistically significant.

## Results

### Natural products library selection and structure characterization

**Fig. 1:**
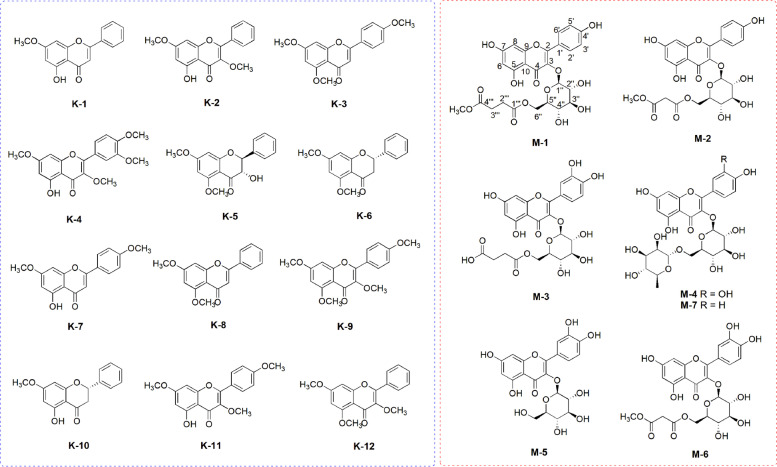
The selected natural products library of flavonoids; the chemical structures of the compounds (**K-1 – K-12**) isolated from *K. parviflora* rhizomes and the compounds (**M-1 – M-7**) from *M. alba* fruits

Flavonoids are natural substances widely found in fruits, vegetables, and plants and represent potential candidates against SARS-CoV-2; the promising results from previous studies suggested that flavonoids can be a vital natural product against SARS-CoV-2 [33]. Among the known anti-SARS-CoV-2 small molecules, flavonoids were prioritized as natural products library in the current study. We initiated our study to discover potential flavonoids as anti-SARS-CoV-2 natural products from our collection of dozens of plants and microorganism-derived extracts assembled through continuous research projects investigating bioactive natural products from diverse natural sources. The LC/MS-based analyses of our extract library, including Global Natural Product Social Molecular Network (GNPS) analysis in combination with our in-house UV library database, revealed that the MeOH extract of black ginger, *Kaempferia parviflora* rhizomes, and the EtOH extract of mulberry (*Morus alba*) fruit were promising samples rich in flavonoids. Phytochemical investigation of the MeOH extract of *K. parviflora* rhizomes and the EtOH extract of *M. alba* fruit using repeated column chromatography and semi-preparative high-performance liquid chromatography (HPLC) separation led to the isolation of 12 methoxyflavones (**K-1 – K-12**) and seven flavonoid glycosides (**M-1 – M-7**) (Fig. 1) from *K. parviflora* rhizomes and *M. alba* fruit, respectively. Structure characterization for the isolated compounds including new compounds (**M-1** and **M-2**) (Table S1, Fig. S1, and Fig. S11-S23) was described in Supplementary Information.

### Cell viability using flavonoids

**Fig. 2:**
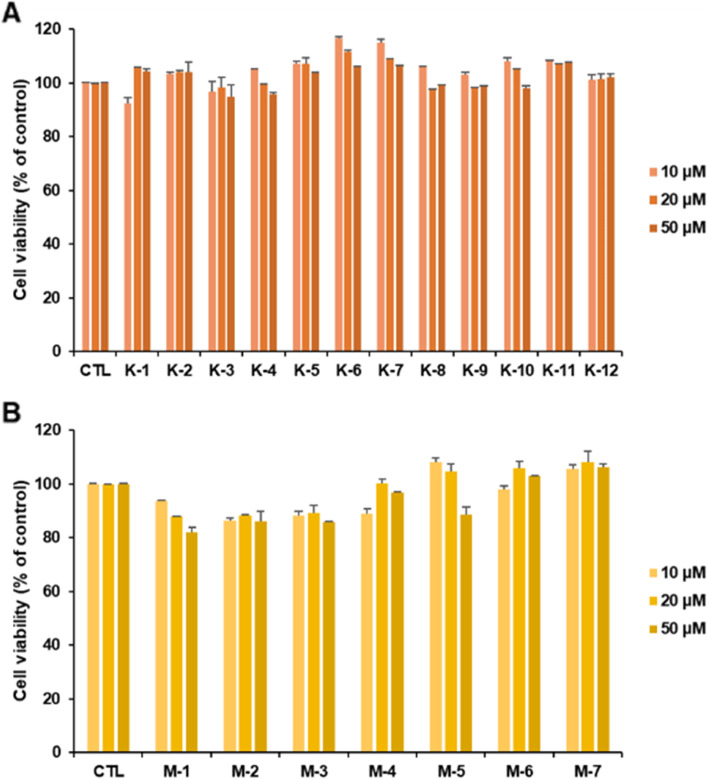
Cell viability of BEAS-2B human bronchial epithelial cells on treatment with K (**A**) and M (**B**) compound series

Cell viability was assessed using MTS assay. BEAS-2B cells were treated with **K** and **M** compound series (10 μM) to investigate whether the compounds exhibited cytotoxic effects on widely used human bronchial epithelial cells. The results suggested that **K** and **M** compound series did not significantly affect cell viability (Fig. 2). Thus, we decided to investigate the antiviral effect of **K** and **M** compound series since these compounds exhibit less cytotoxicity, which is an ideal property for candidate antiviral agents.

### Development of a PV to assess flavonoids-induced degradation of spike protein

We hypothesize that the spike mutations in the Delta or Omicron variant result in the virus’s reduced capacity to utilize ACE2, substantially affecting its entry and replication in A549-ACE2 cells, based on current findings from our experiments and past information [34]. The detailed information on PV production is described in the methods section in the Supplemental data.

**Fig. 3:**
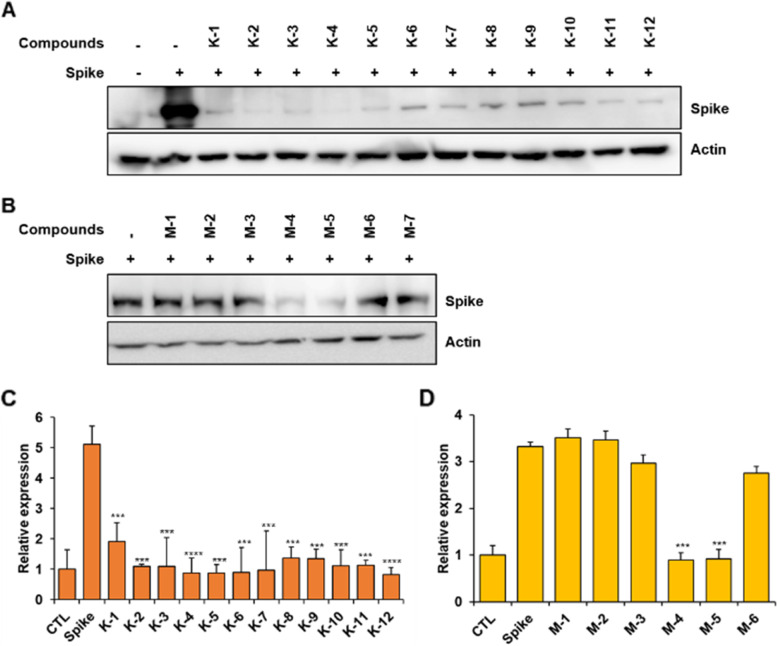
The stability of the Delta-spike protein when treated with different compounds. Delta-spike expression following **K** (**A**) and **M** (**B**) compound series treatment of the cells (10 μM) as analyzed using the Western blot method. Expression levels of the spike protein after the cells were treated with compounds (10 μM) of **K** (**C**) and **M** (**D**) series as determined using whole-cell lysates. Values indicate means ± SEM. (*n* = 3, *** *p* < 0.001, **** < 0.0001 vs. control group)

To evaluate the ability of **K** and **M** compound series to target the spike protein of the pseudotyped-SARS-CoV-2 Delta variant, we evaluated spike expression via western blot following treatment with **K** and **M** compound series (Fig. 3). According to the results, spike expression was markedly downregulated in HEK293T cells due to treatment with **K** and **M** compound series. Most compounds from the **K** series led to a dramatic decrease in spike protein, especially **K-4**. However, only compounds **M-4** and **M-5** from the **M** series resulted in a decreased spike level. Therefore, we tested whether **K-4** or **M-4** had anti-infective properties.

### Inhibition of SARS-CoV-2 variant (Delta and Omicron) or PV entry

**Fig. 4:**
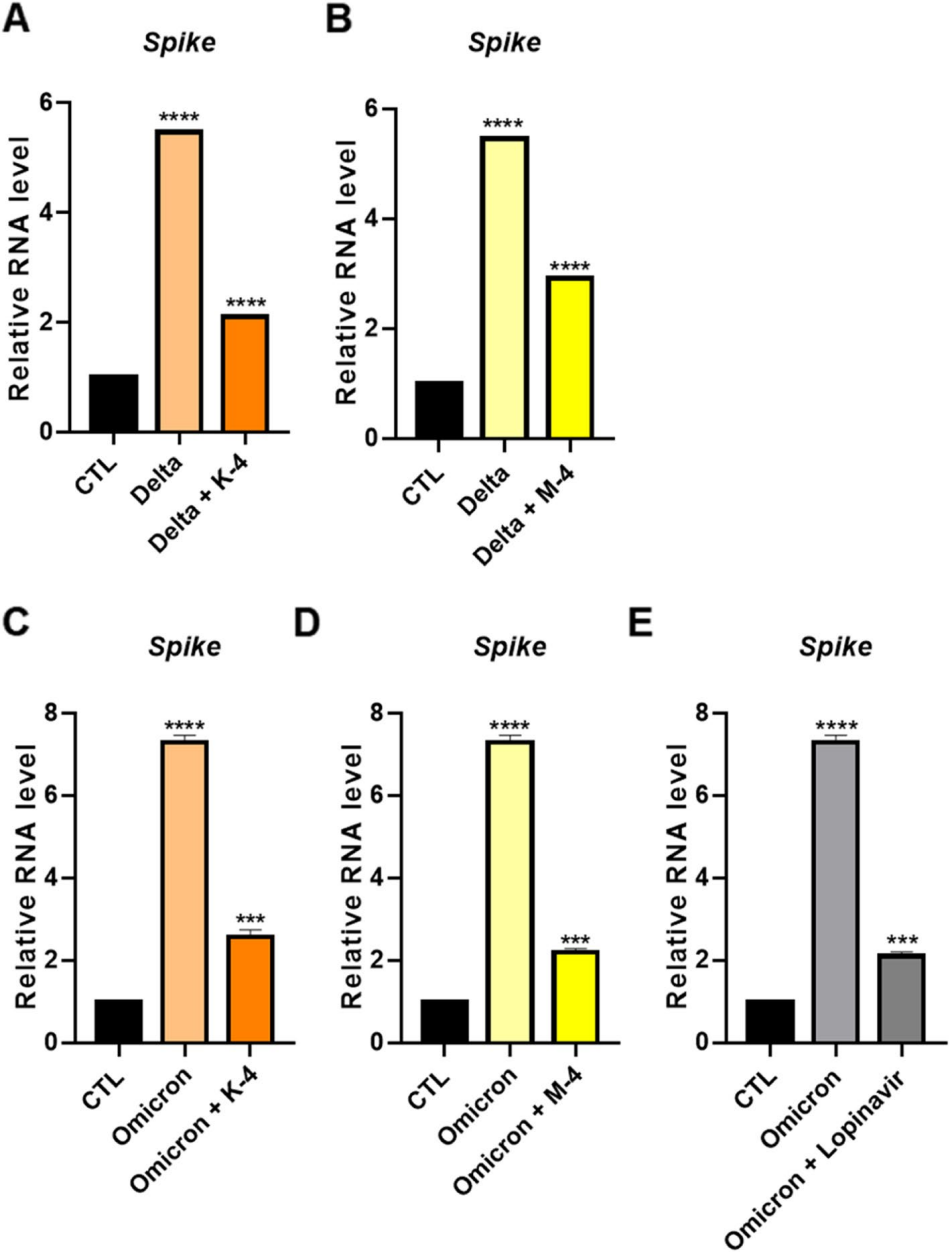
Infectivity tested using compounds of **K-4** and **M-4** (10 μM) against pseudotyped virus (PV) of SARS-CoV-2 Delta (**A**, **B**) or Omicron (**C**, **D**) variant with 10 μM dose of Lopinavir (**E**) as a positive control drug. Values indicate means ± SEM. (*n* = 3, *** *p* < 0.001, **** < 0.0001 vs. control group)

Previous research has shown that lung cells can help investigate PV entry and infection via qPCR analysis of RNA extracts from cell lysates [35]. Infection with pseudotyped SARS-CoV-2 Delta or Omicron spike was performed with equal amounts of input viral load, viral RNA was collected from infected cells at 36 h, and the virus was quantified using RT-qPCR analysis of the spike gene. We found that PVs with Delta or Omicron spikes produced high spike protein RNA copies, which were dramatically reduced after treatment with **K-4** and **M-4** (Fig. 4A-D). These findings demonstrate that **K-4** and **M-4** have strong viral entry capabilities for Delta or Omicron PVs. A previous study reported that lopinavir has the potential for dual-target inhibition, thereby preventing SARS-CoV-2 entry into the host [36]. When we performed an anti-infection assay using lopinavir, PV infection was inhibited effectively, and **K-4** and **M-4** showed similar inhibitory effects (Fig. 4E). Infection with live SARS-CoV-2 Delta or Omicron was performed with equal amounts of input viral load in human bronchial epithelial Calu-3 cells. To confirm the effect of therapeutic efficacy of **K-4** and **M-4**, we performed a plaque assay. Calu-3 cells were pretreated for 4 h with **K-4** or **M-4**, and the cells were exposed to the SARS-CoV-2 Omicron. After 1 h, the virus was removed and plaques were allowed to form. As a result, **K-4** or **M-4** dramatically inhibited plaque formation (Fig. S3A, B). To determine virus levels, viral RNA was collected from infected Calu-3 and the virus was quantified using RT-qPCR analysis of the spike gene. We showed that infection of SARS-CoV-2 Delta or Omicron produced high RNA copies, which were dramatically reduced after treatment with **K-4** and **M-4** (Fig. S3C-F). Thus, we demonstrate that **K-4** and **M-4** are able to significantly reduce SARS-CoV-2 Delta or Omicron infection similar to PV of SARS-CoV-2 variants in cell culture models.

### Anti-inflammatory effect of compounds of K-4 and M-4

**Fig. 5:**
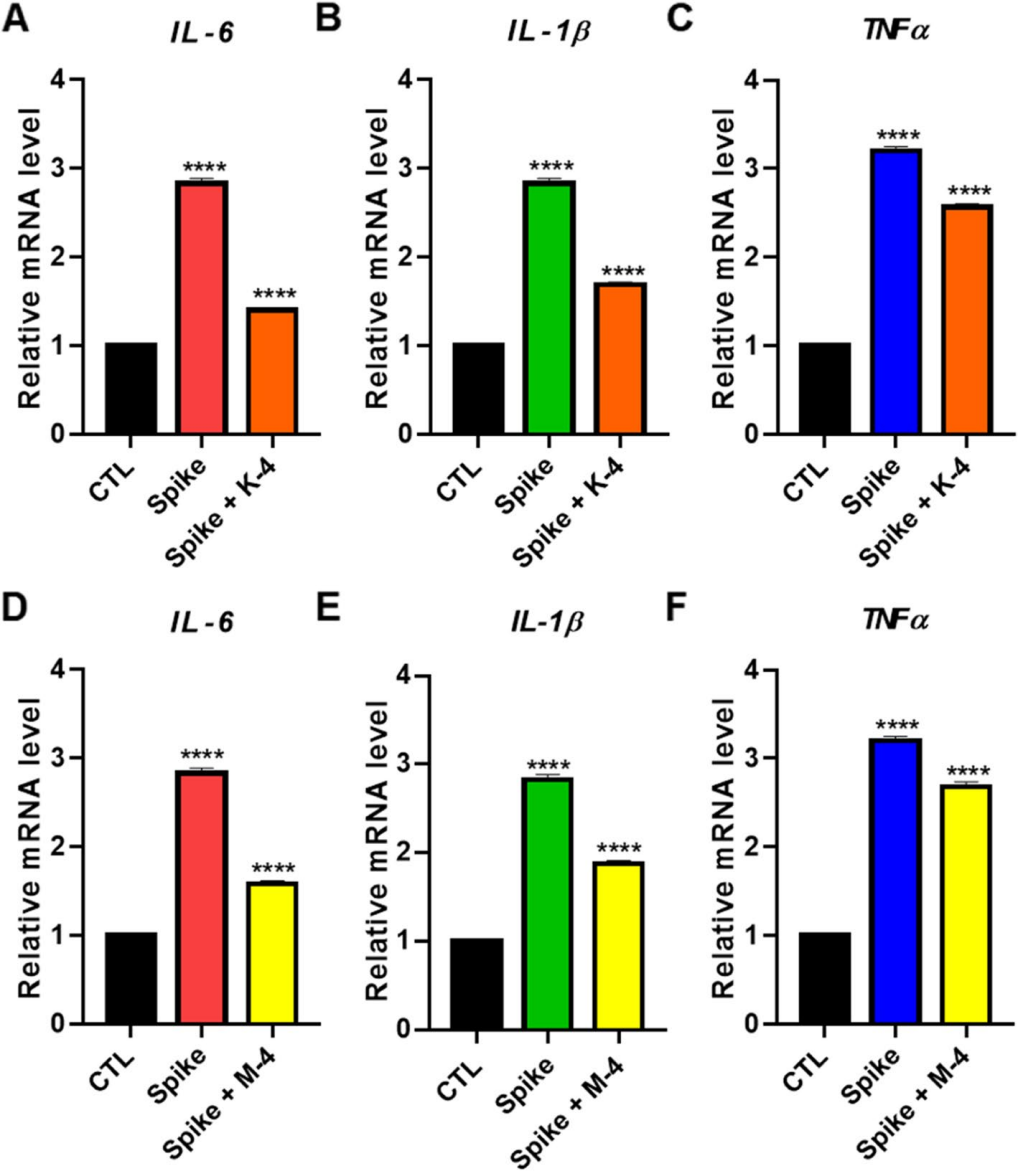
The anti-inflammatory effect of the compounds against Delta-spike protein. A quantitative real-time RT-PCR analysis for IL-6, IL-1β, and TNFα mRNA expression levels after Delta-spike PV infection and **K-4** (**A**, **B**, **C**) or **M-4** (**D**, **E**, **F**) treatment of the cells (10 μM). Values indicate means ± SEM. (*n* = 3, **** < 0.0001 vs. control group)

Previous studies have reported that the SARS-CoV-2 Omicron-B.1.1.529 variant causes a more contagious but less severe disease than the Delta variant strains [37]. Moreover, Omicron shows less pro-inflammatory and IL-6 producing epitopes than other SARS-CoV-2 variants such as Delta [38]. Therefore, we examined the inflammatory effect using Delta-spike PVs. RT-PCR data showed that the titers of all three inflammation-related genes (IL-6, IL-1β, and TNFα) were upregulated upon SARS-CoV-2 Delta-spike PV infection and decreased upon treatment with compounds **K-4** and **M-4** (Fig. 5). These findings demonstrate that the compounds **K-4** and **M-4** have anti-inflammatory effects. In summary, the results indicate that the compounds **K-4** and **M-4** mainly target the SARS-CoV-2 Delta and Omicron spike proteins and thus show potential to be developed into anti-SARS-CoV-2 therapeutics.

### Generation and characterization of lung organoids

**Fig. 6:**
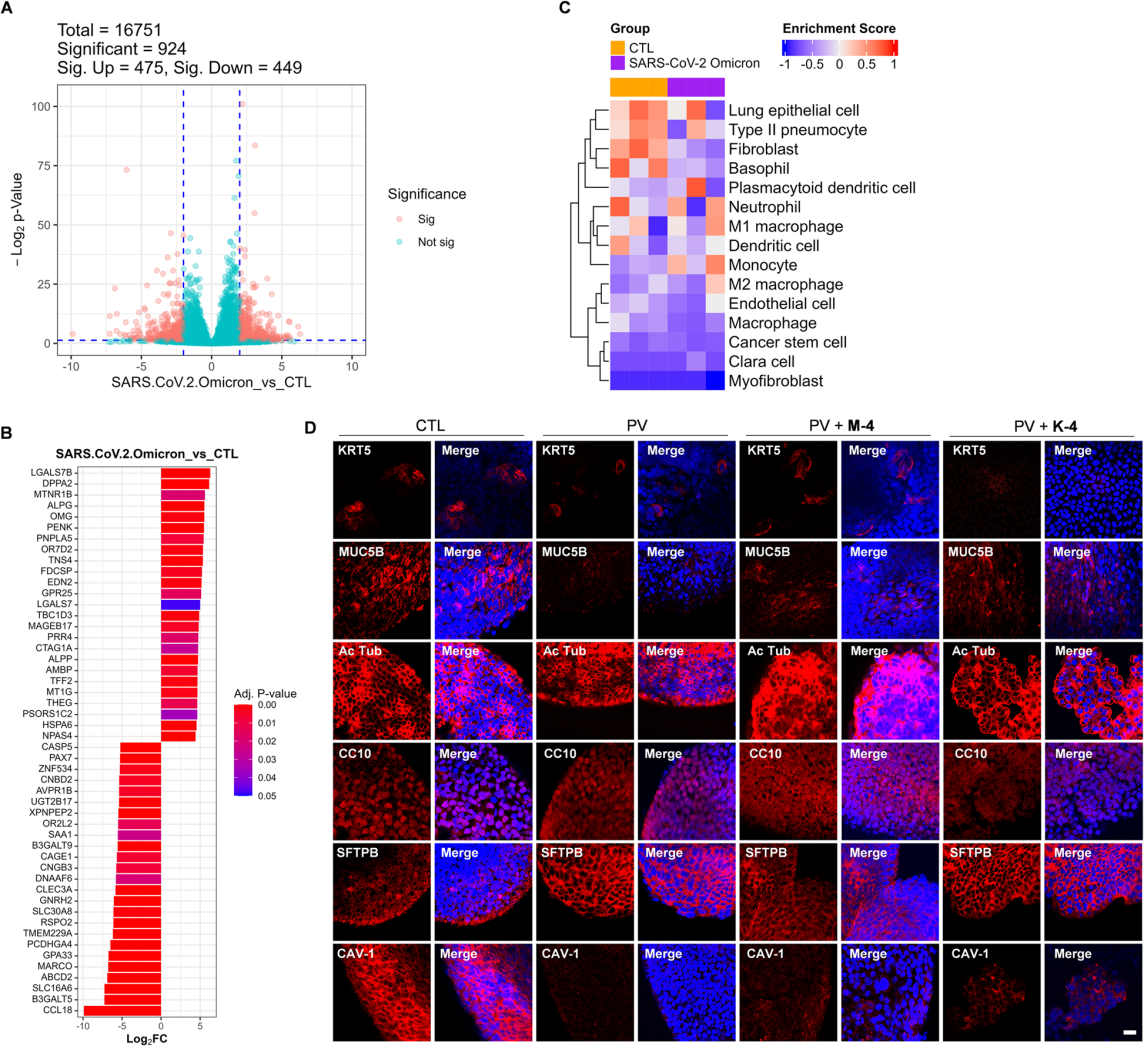
Transcriptome analysis and the cell type analysis in control (GFP) or pseudovirus of SARS-CoV-2 Omicron variant infected in human LOs (*n* = 3). **A** Volcano plot of differentially expressed genes (DEGs) of PV-infected LO samples compared with control samples. Horizontal and vertical dashed blue lines indicate *p*-value < 0.05 and fold change = 4, respectively. Red dots indicate significant (*p*-value < 0.05) DEGs. **B** Top 25 genes of significantly up- or downregulated DEGs. **C** Heatmap of enrichment with lung-related cell types in PV-infected LOs by referring to the CellMarker database. **D** Lung cell-specific markers whole-stained using LOs. LOs were fixed and analyzed with various cell-type markers through immunofluorescence and visualized through confocal microscopy. Scale bar: 20 μm

In previous studies, organoids were primarily used for SARS-CoV-2 infectivity experiments [35]; however, they have never been used for testing the antiviral activity of natural products. To develop a model system for studying the human pulmonary pathophysiology of SARS-CoV-2 infection, we differentiated hiPSCs into LOs following a branching lung organoid protocol with minor modifications [39]. Prior to organoid differentiation, hiPSCs showed higher expression of OCT4 and NANOG in immunofluorescence studies, validating the expression of pluripotency markers (Fig. S2B). First, hiPSCs were differentiated into definitive endoderm (DE), then into anterior foregut endoderm (AFE), lung progenitor cells, and finally into LOs (Fig. S2A, C). We then characterized each differentiation step into specific cell types (Fig. S2D, E). To investigate the effects of the SARS-CoV-2 Omicron variant on the organoids, we performed bulk RNA sequencing to analyze the effect of the PV of SARS-CoV-2 Omicron variant on LOs. As a result of differentially expressed gene (DEG) analysis, the expression levels of 475 genes were significantly increased and the expression levels of 449 genes were significantly decreased (Fig. 6A). Then, we visualized the top and bottom 25 genes based on fold change (Fig. 6B). Next, among the significant DEGs, we checked whether there were ones that matched the genes registered in the SARS-CoV-2-related gene set (Table S2). Significantly upregulated DEGs (Fig. S4A) and downregulated DEGs were visualized as a heatmap (Fig. S4B). As a result, SARS-CoV-2-related gene ontology (GO) terms were displayed.

In addition, we performed subtype variation analysis by referring to the lung-related cell markers using CellMarker database (Table S5) [40]. The heatmap and gene set variation analysis (GSVA) indicated that basophil, fibroblasts, lung epithelial cell, and Type II pneumocyte decreased in PV-infected LOs (Fig. 6C, Fig. S6). We performed immunofluorescence to observe changes in the subtype populations of the PV-infected and **K-4**/**M-4**-treated LOs. The result demonstrated that the major cell markers were expressed in the LOs that showed six cell types, including basal cells (KRT5+), ciliated cells (Ac tub+), goblet cells (MUC5B+), club cells (CC10+), alveolar type II (AT2) cells (SFTPB+), and alveolar type I (AT1) cells (CAV-1) [41, 42]. Therefore, the LOs in our culture are a mixture of many cell types, including alveolar and airway cells. Then, the LOs were infected with the PV of the SARS-CoV-2 Omicron variant. The PV-infected LOs showed cytotoxic effects with reduced marker expression, including the loss of cellular structures (notably, goblet and AT-1 cells). Treatment with **K-4** and **M-4** showed less damage than the PV-infected group (Fig. 6D).

### Anti-viral effects of compounds K-4 and M-4 in the SARS-CoV-2 Omicron spike protein-infected lung organoid models

**Fig. 7:**
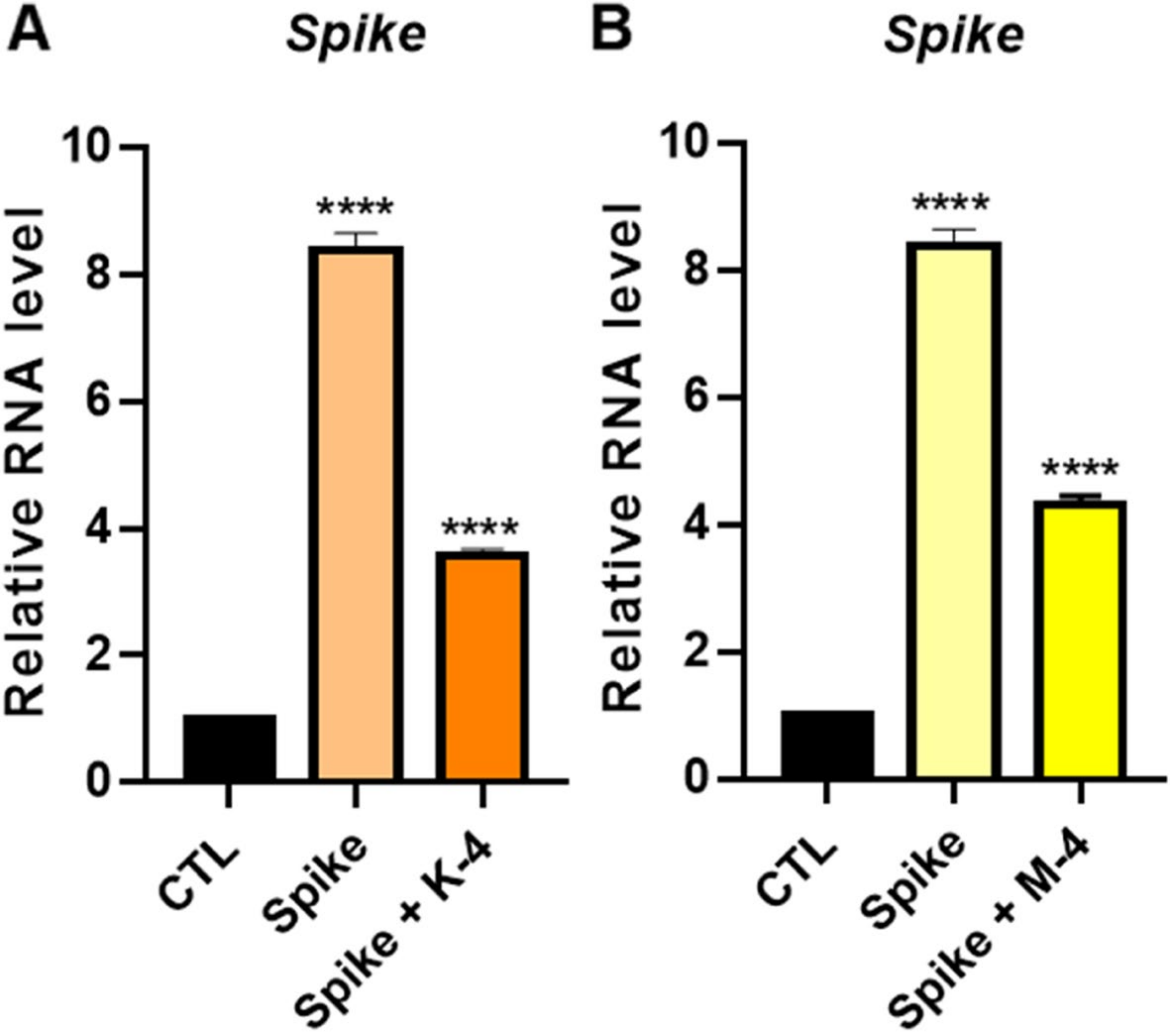
The anti-infective potential of **K-4** (**A**) or **M-4** (**B**) in the lung organoids infected with pseudotyped virus (PV) of SARS-CoV-2 Omicron variant. Values indicate means ± SEM. (*n* = 3, **** < 0.0001 vs. control group)

To evaluate whether LOs were susceptible to SARS-CoV-2 infection, we infected LOs with SARS-CoV-2 Omicron spike PVs to detect viral entry and infection and used GFP signals as a marker. SARS-CoV-2 PVs were successfully incorporated into LOs, as demonstrated by bright-field imaging of the entire LOs merging with robust GFP signals (Fig. S2F). Cells from LOs were lysed to quantify viral infection, and total RNA was isolated. qPCR was used to determine the number of copies of the spike gene in the cells. First, we developed a platform for a SARS-CoV-2 PV-based screening system and confirmed its validity using LOs. Our results conclusively proved that the Omicron spike is markedly transmissible, and treatment with **K-4** or **M-4** dramatically reduced PV infection in the LOs (Fig. 7).

### Molecular docking

**Fig. 8:**
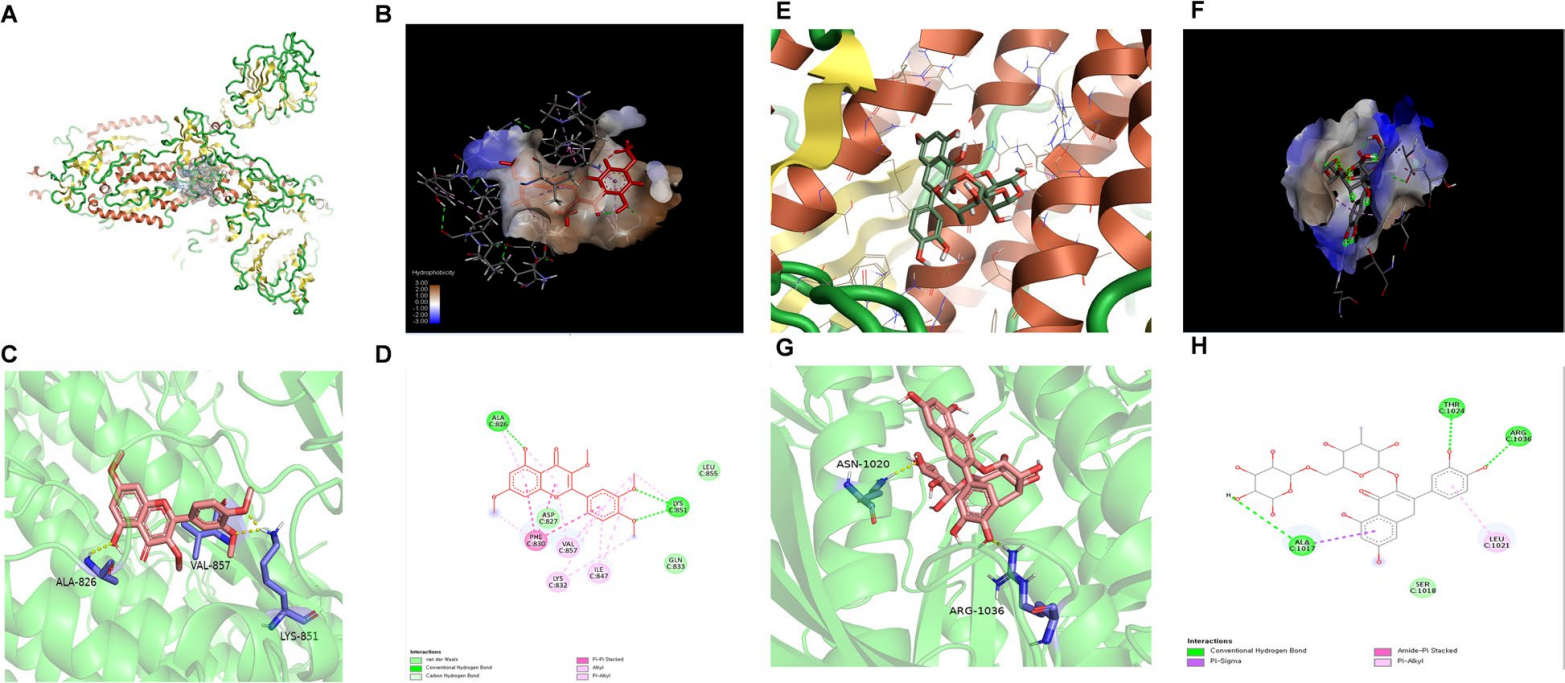
The binding mode of compounds with the spike protein of Omicron variant. Overview of the potential binding pocket for **K-4** (**A**, **B**) and **M-4** (**E**, **F**). The interacting residues are labeled. The residues and ligands are shown in stick figures for **K-4** (**C**) and **M-4** (**G**). The interaction between spike protein and **K-4** (**D**) or **M-4** (**H**) is shown

Based on the structure of the Omicron spike reported we explored the binding mode between **K-4** or **M-4** and the spike protein of the SARS-CoV-2 Omicron variant [32, 43]. **K-4** exhibited low binding energy in the docking study (− 6.5 kcal/mol). The structures of the spike and **K-4** binding pockets are shown (Fig. 8A). According to the result, extensive hydrophobic interactions were observed (Fig. 8B), mediating the stabilization of binding interaction between **K-4** and Omicron spike protein. The 3D and 2D conformations were stabilized by the hydrogen bonds formed between the hydrogen atom of the amine group and the oxygen atom of the ALA826, LYS851, and VAL857 residues (Fig. 8C, D). These results suggest that **K-4** binds to spikes with moderately high binding affinity and may interfere with the stability of the Omicron spike protein. To elucidate the proposed binding mode of **M-4** with the spike protein, Autodock was used to perform molecular docking studies using the crystal structure of **M-4** bound to the core catalytic domain of the Omicron spike protein as a template. **M-4** docked well with the binding site of the spike protein via hydrogen-bonding and T-shaped-stacking interactions (Fig. 8E, F). The glucose group of **M-4** and the hydroxyl moiety on the benzene ring portions were deeply nested within the catalytic cave of the Omicron spike protein, which was immobilized by two H-bonds. One H-bond was formed by the OH group of the glucose moiety bound to residue ASN1020, and the other was formed via the hydroxy group on the benzene rings conjugated to residue ARG1036. The C ring of the flavonoid structure was stretched out of the catalytic pocket of the Omicron spike protein and formed a T-shaped π–π stacking interaction with the LEU1021 residue as expected to occupy one peripheral groove (Fig. 8G, 8H). SARS-CoV-2 enters cells by cleaving its spike protein using the transmembrane protease serine subtype 2 (TMPRSS2) and ACE2 receptor [43]. Molecular docking studies were performed to identify the critical binding regions of the active flavonoids (**K-4** and **M-4**) with spike or ACE2. Our results strongly suggest that **K-4** or **M-4** binds to spike and inhibits the interaction between spike and ACE2 to block virus infection.

## Discussion

Natural products are a source of antiviral agents that may still be untapped. The secondary metabolites found in plants are notable for their ability to protect plants. Polyphenols are a common type of secondary metabolites, and flavonoids are one of the main polyphenol classes [44]. The flavonoid paradox claims that many flavonoids have been shown to have therapeutic effects for various pathologies in vivo [45]. The main protease of the SARS-CoV-2 virus is targeted frequently for developing antivirals [46]. Particularly, several polyphenols demonstrated strong antiviral activity against the main protease of the SARS-CoV-2. Among these, the polyphenolic flavonoid was unique for its ability to inhibit the main protease activity in a cell-based assay effectively [47]. Flavonoids have been proposed to interfere with the coronavirus life cycle at the stages of penetration and entry of the viral particle into the cell, replication of the viral nucleic acid, and release of the virion from the cell [48, 49]. Flavonoids with antiviral activities against SARS-CoV-2 have been targeted mainly against 3CLpro, followed closely by disrupting the S-ACE2 interaction and PLpro [50]. The peptide chain processing reaction depends on the enzymes 3CLpro and PLpro. They cleave the polypeptide chain’s C-terminus at 11 different locations and its N-terminus at 3 different locations. Structure-related proteins and certain crucial non-structural proteins, such RNA-dependent RNA polymerase (RdRp) and helicase, are among the cleavage products [51]. Quercetin and rutin (quercetin-3-*O*-rutinoside) were reported to possess anti-SARS-CoV-2 3CLpro activities, acting as competitive inhibitors at the 3CLpro active site [52]. Another promising target for anti-SARS-CoV-2 therapeutic intervention is the interaction between the viral S protein and the human ACE2 receptor during the viral entry phase [53, 54]. Quercetin and its derivatives as well as epigallocatechin gallate (EGCG) are the flavonoids most representative of in vitro inhibitory activities against SARS-CoV-2 spike RBD–ACE2 interaction [53–55]. Thus, we tried developing different approaches to identify the dual function of these flavonoids, i.e., decreasing the spike stability when SARS-CoV-2 has already infected or inhibiting the infection at the beginning of the infection cycle.

Most efforts to develop COVID-19 therapeutics aim at either decreasing the stability or exploiting some of the structural features of the S protein, which triggers immune responses and plays a vital role in the ability of the virus to infect the host. The furin cleavage allows S1 RBDs and N-terminal domains (NTDs) to move more freely, resulting in lower thermal stability and more receptor binding-competent forms of spike protein [56].

The binding of spike and ACE2 receptors induces a cascade of inflammatory and cytokine storms, and the virus is internalized [57]. Considering these factors, an effective therapeutic strategy may involve targeting the ACE2 to prevent the interactions of the Spike protein of SARS-CoV-2 with the receptor, thereby preventing viral infection and the inflammatory storm [58]. We believe that compounds **K-4** and **M-4** will ultimately help suppress the lung inflammation caused by SARS-CoV-2 infection.

To study the biology of SARS-CoV-2 and to facilitate drug screening, novel models utilizing human disease-relevant cells are urgently required. Organoids, including those of infectious diseases, have been used as models for a wide range of pathologies [59]. Recently, a few studies utilized LOs to study SARS-CoV-2 inhibitors such as FDA-approved drugs [60]. However, to the best of our knowledge, no study has so far tested SARS-CoV-2 therapeutic natural products using lung organoid models. Moreover, the docking results supported the anti-infectivity effects of **K-4** and **M-4** in the LOs. Further molecular docking studies are needed to determine how flavonoids regulate spike protein stability.

After selecting the docking grid box for 7TGW, we found a protein cavity that may be a potential docking pocket near residue S979, a new mutation residue in the Omicron spike. Here we considered the existence of so-called “attracting cavities” and performed molecular docking only by energy minimization. After screening, we selected the docked confirmation with the highest score. The ligand had the lowest affinity energy, probably due to its deeper placement in the protein cavity. Figure 8E visually shows the location of the ligand docks with the protein and the conformation of the ligand in the active pocket. Figure 8F shows that ligands exhibit hydrophobic interactions with cavity-forming residues. The darker the blue, the stronger the hydrophobic force. Hydrophobic interactions are attractive short-range interactions that contribute to ligand-receptor binding affinities. During visualization with PyMOL and Discovery Studio, we found that two hydrogen bonds were formed with residues ASN1020 and ARG1036 as the ligands become more stable in the cavity during hydrogen bond formation, resulting in a decrease in entropy. At the same time, there were other intermolecular forces between the ligand and the amino acid residues ALA1017, SER1018, and LEU1021 to enhance the binding force of the ligand. This reasonably explains why this conformation exhibited the lowest binding energy. The biological role of these amino acid residues on the overall protein needs to be further explored; they may hinder the conformational transition of the protein configuration, thereby reducing the protein’s activity.

In a recent paper [61], we were able to find analysis results obtained from SARS-CoV-2 patients and non-virally infected controls. Here, the list of genes upregulated in the patient was presented (Table S3) and a visual representation is presented describing how genes were expressed in SARS-CoV-2 Omicron variant PV-infected LOs (Fig. S5A, Table S4). Moreover, we also performed gene ontology (GO) analysis of the up and downregulated genes between the two groups (Fig. S5B). GO terms associated with decreased DEG in the PV-infected LOs were related to oxidative stress, cytokine production in the inflammatory response, and interleukin-6 response. In addition, GO terms associated with increased DEG in the PV-infected LOs were related to immunity, apoptosis, and various stress response. Gene set variation analysis (GSVA) was also performed to quantify the genetic change tendency, and as a result, it was confirmed that there was a significant difference between the SARS-CoV-2 Omicron variant PV-infected LOs and the control group (Fig. S5C).

In addition, a recent study included various lung cell line analysis results in addition to patient samples [62]. We performed GSVA between the gene sets from lung cell lines and SARS-CoV-2 Omicron variant PV-infected LOs. As a result of comparing and quantifying through GSVA [63], it was confirmed that there was a significant difference between the control group and the SARS-CoV-2 Omicron variant PV-infected LO group (Fig. S7A, S7C). Based on the upregulated genes in patient tissue (Table S6), 54 genes are downregulated and clustered in leukocyte, lymphocyte, macrophage migration, and chemotaxis. Another 54 genes are upregulated and clustered in cytokine-mediated signaling pathways, innate immune response, T cell differentiation, and interleukin production (Fig. S7B). A recent paper described how their group performed single-nucleus RNA sequencing from the lungs of 19 individuals who died of COVID-19 and performed rapid autopsies with seven control individuals [64]. Due to the loss of both alveolar type II (AT2) and type I (AT1) cells, there was a reduction in the epithelial cell compartment, while monocytes/macrophages, fibroblasts, and neuronal cells increased in SARS-CoV-2-infected lungs. A limitation of lung organoid models is that they do not have immune cell co-culture conditions; however, further single-cell sequencing analysis would be important to compare between SARS-CoV-2 Omicron variant PV-infected LOs and SARS-CoV-2-infected human lung tissue. There are some other limitations of studies that use PVs of SARS-CoV-2 systems. PVs of SARS-CoV-2 only contain the spike protein of the SARS-CoV-2 Omicron variant. Also, PVs cannot replicate in host cells. For these reasons, PVs of SARS-CoV-2 may not always change subtype populations or promote pathogenesis as much as live SARS-CoV-2. On the other hand, the advantage of PVs of SARS-CoV-2 systems is that we can monitor specific single viral protein effects, such as effects relating to the spike protein of SARS-CoV-2 (Fig. 6D).

We have already shown the anti-viral effects of the compounds **K-4** and **M-4** in SARS-CoV-2 Omicron spike protein-infected lung organoid models (Fig. 7), and the mechanism of action was proven by molecular docking results: compounds bind to the spike and inhibit the interaction between the spike and ACE2 to block virus infection. Several papers have reported similar mechanisms of action of small-molecule agents [65–68]. Also, the potential molecular mechanisms of the anti-viral effects were investigated in LOs. To illustrate the interactions between the DEGs and the enriched Kyoto Encyclopedia of Genes and Genomes (KEGG) pathways, up- or downregulated DEGs are visualized in the COVID-19 pathway (Fig. S8 and Table S7). Pathway-related GO analysis revealed that the chemokine-mediated signaling pathway was significantly upregulated in PV-infected LOs (Fig. S9A). The expression levels of *TFF2*, *PF4*(*CXCL4*), *XCL1*, *CXCR6*, *CXCL1*, *CXCL6*, *CXCL2*, *CCL2*, and *CXCL8* were upregulated in PV-infected LOs (Fig. S9B and Table S8). Chemokines are crucial inflammatory mediators required during an immune response to clear pathogens [69, 70]. Therefore, another possible mechanism of anti-viral effects is reducing inflammation.

In addition, a conventional disease ontology (DO) analysis was performed based on a database of disease-related genes (Table S9) and visualized with a dot plot (Fig. S10). As a result of the DO analysis on the significant DEGs, it was found that lung disease terms such as lung disease and pulmonary hypertension were ranked at the top above SARS-CoV-2 Omicron variant PV-infected LOs.

To the best of our knowledge, there are several promising active natural products that may provide targeted anti-COVID-19 drugs that are undergoing preclinical or clinical studies [71–73]. It would be interesting to investigate the therapeutic efficacy of **K-4** and **M-4** under preclinical or clinical studies as well. In addition, it will be important to test SARS-CoV-2 therapeutic natural products using lung organoid models to select potential antiviral drugs.

## Conclusions

More small-molecule medicines should be developed and kept ready for present and potential future viruses, as it is anticipated that SARS-CoV-2 will continue affecting people over the coming years [74]. Numerous natural products, including flavonoid family members with good antiviral activity, have been developed. However, the development of a wide range of medicines to treat current coronaviruses, including SARS-CoV-2, could be made possible by using the new screening platform [75]. In this study, we established an infection model using a pseudotyped virus in both cell lines and organoids. We developed a robust screening platform to determine the dual function of inhibiting infection at the start of the infection cycle and reducing spike stability if the SARS-CoV-2 infection has already been initiated. Using this platform, we found that flavonoids **K-4** and **M-4** in our natural product library had the biological ability to inhibit the infection and spike stability of SARS-CoV-2 variant PV. Moreover, **K-4** and **M-4** could effectively reduce the inflammation caused by the virus and reduce the expression of pro-inflammatory mRNAs, such as IL-6, IL-1β, and TNFα. Thus, **K-4** and **M-4** could potentially be developed as antiviral drugs, and the screening platform developed can be a standard system to discover novel drug candidates against SARS-CoV-2.

## Supplementary Information

**Additional file 1.**

**Additional file 2.**

**Additional file 3.**

**Additional file 4.**

**Additional file 5.**

**Additional file 6.**

**Additional file 7.**

**Additional file 8.**

**Additional file 9.**

**Additional file 10.**

## Data Availability

The datasets used and/or analysed during the current study are available from the corresponding author on reasonable request.

## Abbreviations

COVID-19: Coronavirus disease 2019
PCR: polymerase chain reaction
RT-PCR: Reverse transcription PCR
RT-qPCR: Reverse transcription quantitative PCR
NGS: Next-generation sequencing
SARS-CoV-2: Severe acute respiratory syndrome coronavirus-2
LOs: lung organoids
hiPSCs: Human induced pluripotent stem cells
BSL-3: biosafety level 3
PVs: pseudotyped viruses
ACE2: angiotensin-converting enzyme 2
ORF: open reading frame
nsps: nonstructural proteins
S: spike
RBD: receptor-binding domain
nAbs: neutralizing antibodies
VSV: vesicular stomatitis virus
3CLpro: 3-chymotrypsin-like protease
PLpro: papain-like protease
RdRp: RNA-dependent RNA polymerase
DMEM: Dulbecco’s modified Eagle’s medium
RPMI: Roswell Park Memorial Institute
FBS: fetal bovine serum
SDS-PAGE: sodium dodecyl sulfate-polyacrylamide gel electrophoresis
BSA: bovine serum albumin
GNPS: Global Natural Product Social Molecular Network
HPLC: high-performance liquid chromatography
MTS: 3-(4,5-dimethylthiazol-2-yl)-5-(3-carboxymethoxyphenyl)-2-(4-sulfophenyl)-2H-tetrazolium
DE: definitive endoderm
AFE: anterior foregut endoderm
TMPRSS2: transmembrane protease serine subtype 2
EGCG: epigallocatechin gallate
NTDs: N-terminal domains
DEG: differentially expressed gene
GO: gene ontology
GSVA: Gene set variation analysis
AT1: alveolar type I
AT2: alveolar type II
KEGG: Kyoto Encyclopedia of Genes and Genomes
DO: disease ontology
