# Supplementary material for: Development of a screening platform to discover natural products active against SARS-CoV-2 infection using lung organoid models
Source: Biomater Res. 2023 Mar 1;27:18. doi: 10.1186/s40824-023-00357-y (PMC9974403; doi:10.1186/s40824-023-00357-y)
Supplement: Supplementary file 1 — Additional file 1. [file 40824_2023_357_MOESM1_ESM.docx]

Development of a screening platform to discover natural products active against SARS-CoV‑2 infection using lung organoid models

Joo-Eun Lee^1,a^, Se Yun Jeong^2,a^, Zijun Li^1,3,a^, Hyun-Yi Kim^4^, Hyun-Woo Kim^5^, Min Jeong Yoo^2^, Hee Joo Jang^2^, Do-Kyun Kim^5^, Namki Cho^1^, Hee Min Yoo^3,6,^* ,Ki Hyun Kim^2,^*

^1^ *College of Pharmacy, Chonnam National University, Gwangju, 61186, Republic of Korea*

^2^ *School of Pharmacy, Sungkyunkwan University, Suwon 16419, Republic of Korea*

^3^ *Biometrology Group, Korea Research Institute of Standards and Science (KRISS), Daejeon 34113, Republic of Korea*

^4^ *NGeneS Inc., Ansan 15495, Republic of Korea.*

^5^ *Korea Zoonosis Research Institute, Jeonbuk National University, Iksan 54531, Republic of Korea*

^6^ *Department of Precision Measurement, University of Science and Technology (UST), Daejeon 34113, Republic of Korea*

^a^ These authors contributed equally to this work

*** Corresponding authors:

Ki Hyun Kim^1^, Tel: +82-31-290-7700; Fax: +82-31-290-7730; E-mail: khkim83@skku.edu

Hee Min Yoo^3,6^, E-mail: hmy@kriss.re.kr

Table of Contents

**Supplemental Methods**

**Table S1.** ^1^H (850 MHz) and ^13^C NMR (212.5 MHz) data for compounds **M-1** and **M-2** in CD_3_OD (δ ppm)

**Natural Products Library Selection and Structure Characterization**

**Figure S1.** ^1^H−^1^H COSY ( ) and key HMBC () correlations of compounds **M-1** and **M-2**.

**Figure S2.** Modeling SARS-CoV-2 infection using hiPSC differentiation into lung organoids (LOs).

**Figure S3**. Inhibition of SARS-CoV-2 variant infection of Calu-3 cells.

**Figure S4**. The enriched gene sets in control (GFP) or pseudovirus of SARS-CoV-2 Omicron variant infected in human LOs**.**

**Figure S5**. Differentially expressed gene (DEG) and gene ontology (GO) analyses of transcriptome in the control (GFP) and SARS-CoV-2 Omicron variant pseudovirus-infected human LOs.

**Figure S6**. The subtype variation analysis.

**Figure S7**. Differentially expressed gene (DEG), gene ontology (GO), and gene set variation analysis (GSVA) of PV-uninfected or infected LOs on gene sets of the COVID-19 patient samples.

**Figure S8**. Kyoto Encyclopedia of Genes and Genomes (KEGG) pathway and differentially expressed genes (DEGs)

**Figure S9**. Gene ontology (GO) and differentially expressed gene (DEG) of PV-uninfected or infected LOs.

**Figure S10**. Disease Ontology (DO) enrichment analyses.

**HR-MS, and 1D- and 2D-NMR data for compounds M-1 and M-2**

**Figure S11.** HR-ESI-MS of compound **M-1**

**Figure S12.** ^1^H NMR spectrum of compound **M-1**

**Figure S13.** HSQC spectrum of compound **M-1**

**Figure S14.** ^1^H−^1^H COSY spectrum of compound **M-1**

**Figure S15.** HMBC spectrum of compound **M-1**

**Figure S16.** NOESY spectrum of compound **M-1**

**Figure S17.** Retention time of reaction product of thiocarbamoyl-thiazolidine derivative of glucose (A) from compound **M-1** and β-ᴅ-glucose standard (B)

**Figure S18.** HR-ESI-MS of compound **M-2**

**Figure S19.** ^1^H NMR spectrum of compound **M-2**

**Figure S20.** HSQC spectrum of compound **M-2**

**Figure S21.** ^1^H−^1^H COSY spectrum of compound **M-2**

**Figure S22.** HMBC spectrum of compound **M-2**

**Figure S23.** NOESY spectrum of compound **M-2**

**Supplemental References**

**Supplemental Methods**

*General experimental procedures*

Optical rotations were measured using a P-2000 polarimeter (JASCO, Easton, MD, USA). Ultraviolet (UV) spectra were acquired using Agilent 8453 UV-visible spectrophotometer (Agilent Technologies, Santa Clara, CA, USA). The ECD spectra were obtained using a JASCO J-1500 spectropolarimeter (JASCO). Infrared (IR) spectra were recorded using a Bruker IFS-66/S FT-IR spectrometer (Bruker, Karlsruhe, Germany). NMR spectra were recorded with a Bruker AVANCE III HD 850 NMR spectrometer with a 5 mm TCI CryoProbe operating at 850 MHz (^1^H) and 212.5 MHz (^13^C), with chemical shifts given in ppm (δ) for ^1^H and ^13^C NMR analyses. All HR-ESIMS data were obtained using an Agilent G6545B quadrupole time-of-flight mass spectrometer (Agilent Technologies) coupled to an Agilent 1290 Infinity II HPLC instrument using an Agilent Eclipse Plus C18 column (2.1 × 50 mm, 1.8 μm; flow rate: 0.3 mL/min). Preparative HPLC was performed using a Waters 1525 Binary HPLC pump with a Waters 996 Photodiode Array Detector (Waters Corporation, Milford, MA, USA) and an Agilent Eclipse C18 column (250 × 21.2 mm, 5 μm; flow rate: 5 mL/min; Agilent Technologies). Semi-preparative HPLC was performed using a Shimadzu Prominence HPLC System with SPD-20 A/20AV Series Prominence HPLC UV-Vis detectors (Shimadzu, Tokyo, Japan) and Phenomenex Luna C18 column (250 × 10 mm, 5 μm; flow rate: 2 mL/min; Phenomenex, Torrance, CA, USA). LC/MS analysis was performed on an Agilent 1200 Series HPLC system equipped with a diode array detector and 6130 Series ESI mass spectrometer using an analytical Kinetex C18 100 Å column (100 × 2.1 mm, 5 μm; flow rate: 0.3 mL/min; Phenomenex). Silica gel 60 (230–400 mesh; Merck, Darmstadt, Germany) and RP-C18 silica gel (230–400 mesh; Merck) were used for column chromatography. Sephadex LH-20 (Pharmacia, Uppsala, Sweden) was used as the packing material for the molecular sieve column chromatography. Thin-layer chromatography (TLC) was performed using pre-coated silica gel F254 plates and RP-C18 F254s plates (Merck), and spots were detected under UV light or by heating after spraying with anisaldehyde-sulfuric acid.

*Sample materials*

*Kaempferia parviflora* rhizomes were collected in January 2020 from Chiang Mai City, northern Thailand. This material was authenticated by one of the authors (K.H.K). A voucher specimen (SKKU-BG 1908) was stored in the herbarium at the School of Pharmacy, Sungkyunkwan University, Suwon, Korea. *Morus alba* fruits were purchased from the Kyungdong Market (Woori Herb), Seoul, Korea, in January 2014. This material was authenticated by K. H. Kim. A voucher specimen of the material (MA 1414) was deposited in laboratory 306 at the Dong-A ST Research Center, Yongin, Korea.

*Extraction and isolation of natural products*

*Isolation of natural products from K. parviflora rhizomes*

Dried *K. parviflora* rhizomes (132 g) were crushed and then extracted with 80% MeOH/H_2_O (2.0 L, 24 h × 2) at room temperature, and then the residue of *K. parviflora* rhizomes was extracted with 80% MeOH/H_2_O (3.0 L, 12 h) under reflux. The filtered extracts were combined and evaporated under reduced pressure using a rotary evaporator to obtain a crude MeOH extract (9.2 g). The resultant extract was suspended in distilled water (700 mL), and solvent partitioning of the extract was performed using *n*-hexane (700 mL), dichloromethane (CH_2_Cl_2_, 700 mL), ethyl acetate (EtOAc, 700 mL), and *n*-butanol (*n*-BuOH, 700 mL), three times. Four fractions with increasing polarity, namely, the hexane-soluble (1.0 g), CH_2_Cl_2_-soluble (3.2 g), EtOAc-soluble (0.4 g), and *n*-BuOH-soluble (0.5 g) layers, were obtained. With reference to an in-house UV library, LC-MS analysis of the four fractions derived from the solvent partition process confirmed the presence of the majority of flavonoids in the hexane-soluble fraction. The hexane-soluble fraction (1.0 g) was subjected to silica gel column chromatography (eluted with *n*-hexane/EtOAc [10:1 → 1:1] and CH_2_Cl_2_/MeOH [10:1→1:1], gradient solvent system) to obtain six fractions (H1–H6). Fraction H1 (31.1 mg) was separated by semi-preparative reverse-phase HPLC with 94% MeOH/H_2_O (flow rate of 2 mL/min) to obtain compound **K-10** (2.3 mg, *t*_R_ = 14.5 min). Fraction H2 (91.5 mg) was separated using Sephadex LH-20 column chromatography [CH_2_Cl_2_/MeOH (2:8)] to obtain five subfractions (H21–H25). Subfraction H22 (29.2 mg) was isolated using semi-preparative reverse-phase HPLC with 78% MeOH/H_2_O (flow rate of 2 mL/min) to obtain compounds **K-1** (4.1 mg, *t*_R_ = 35.5 min) and **K-2** (3.9 mg, *t*_R_ = 39.8 min). Fraction H4 (65.8 mg) was purified using semi-preparative reverse-phase HPLC with 70% MeOH/H_2_O (flow rate of 2 mL/min) to yield compounds **K-5** (1.2 mg, *t*_R_ = 16.8 min) and **K-8** (2.0 mg, *t*_R_ = 21.3 min). Fraction H5 (112.7 mg) was subjected to Sephadex LH-20 column chromatography [CH_2_Cl_2_/MeOH (2:8)] to obtain two subfractions (H51 and H52). Subfraction H52 (28.2 mg) was separated using semi-preparative reverse-phase HPLC with 83% MeOH/H_2_O (flow rate of 2 mL/min) to isolate compounds **K-4** (3.8 mg, *t*_R_ = 26.3 min), **K-7** (2.5 mg, *t*_R_ = 28.1 min), and **K-11** (0.6 mg, *t*_R_ = 31.5 min). Fraction H6 (271.8 mg) was fractionated using Sephadex LH-20 column chromatography (CH_2_Cl_2_/MeOH [2:8]) to obtain three subfractions (H61–H63). Subfraction H61 (14.5 mg) was isolated using semi-preparative reverse-phase HPLC with 42% MeCN/H_2_O (flow rate of 2 mL/min) to obtain compounds **K-6** (2.8 mg, *t*_R_ = 40.2 min), **K-3** (0.8 mg, *t*_R_ = 44.0 min), and **K-9** (0.5 mg, *t*_R_ = 45.9 min). Subfraction H62 (228.5 mg) was subjected to silica gel column chromatography (eluted with CH_2_Cl_2_/MeOH [60:1→1:1], gradient solvent system) to obtain six subfractions (H621–H626). Subfraction H622 (20.1 mg) was purified using semi-preparative reverse-phase HPLC with 76% MeOH/H_2_O (flow rate of 2 mL/min) to obtain compound **K-12** (1.4 mg, *t*_R_ = 47.2 min).

*Isolation of natural products from Morus alba fruits*

Dried and mashed *M. alba* fruits (1.1 kg) were extracted by mixing the material with 70% aqueous ethanol (EtOH) (15.0 L) for three days at room temperature; this was repeated three times, followed by filtration. After evaporation of the filtrate *in vacuo*, the resultant residue (120 g) was dissolved in distilled water (800 mL) and then solvent-partitioned with *n*-hexane (800 mL), chloroform (CHCl_3_, 800 mL), ethyl acetate (EtOAc, 800 mL), and *n*-butanol (*n*-BuOH, 800 mL), three times. Four layers with increasing polarity, hexane-soluble (2.8 g), CHCl_3_-soluble (8.5 g), EtOAc-soluble (3.2 g), and *n*-BuOH-soluble (13.8 g) fractions, were obtained. LC-MS/MS analysis of the solvent-partitioned fractions obtained in combination with our in-house UV library database revealed that the EtOAc-soluble fraction was rich in flavonoids. The EtOAc-soluble fraction (3.2 g) was fractionated by RP-C_18_ column chromatography (eluted with MeOH/H_2_O [30:70 → 60:40 → 100:0], gradient solvent system) to obtain seven fractions (E1–E7). Fraction E5 (0.5 g) was further separated on a Sephadex LH-20 column using a solvent system of 100% MeOH to yield seven subfractions (E51-E57). Subfraction E55 (150 mg) was fractionated by preparative reversed HPLC (eluted with MeOH/H_2_O [30:70 → 80:20] in 120 min, gradient solvent system) to yield four subfractions (E551-E554). Subfraction E553 (25.3 mg) was purified using semi-preparative HPLC with 30% MeOH/H_2_O (flow rate of 2 mL/min) to obtain compounds **M-4** (3.5 mg, *t*_R_ 30.0 min), **M-5** (0.6 mg, *t*_R_ 33.8 min), and **M-7** (1.5 mg, *t*_R_ 48.2 min). Subfraction E56 (50 mg) was purified using semi-preparative HPLC with 45% MeOH/H_2_O (flow rate of 2 mL/min) to afford compounds **M-3** (1.7 mg, *t*_R_ 38.9 min) and **M-6** (2.2 mg, *t*_R_ 54.0 min). Fraction E6 (0.4 g) was separated on a Sephadex LH-20 column using a solvent system of 100% MeOH to yield five subfractions (E61–E65). Subfraction E63 (19.8 mg) was purified using semi-preparative HPLC with 53% MeOH/H_2_O (flow rate of 2 mL/min) to afford compounds **M-1** (1.1 mg, *t*_R_ 54.1 min) and **M-2** (0.8 mg, *t*_R_ 44.9 min).

*Kaempferol-3-O-glucoside 6"-succinate methyl ester (****M-1****)*

Yellowish amorphous powder; [α]-19.0 (*c* 0.05, MeOH); UV (MeOH) *λ*_max_ (log ε) 250 (3.8), 329 (3.5) nm; IR (KBr) ν_max_ 3420, 2935, 2365, 1658, 1560, 1510, 1462, 1025 cm^-1^; ^1^H and ^13^C NMR (850 and 212.5 MHz, respectively) (Table S1); negative high-resolution electrospray ionization mass spectroscopy (HR-ESIMS) *m/z* 561.1248 [M - H]^-^ (calculated for C_26_H_25_O_14_, 561.1244).

*Kaempferol-3-O-glucoside 6"-malonate methyl ester (****M-2****)*

Yellow amorphous powder; [α]-15.0 (*c* 0.04, MeOH); UV (MeOH) *λ*_max_ (log ε) 246 (3.8), 328 (3.5) nm; IR (KBr) ν_max_ 3415, 2938, 2364, 1655, 1561, 1510, 1460, 1025 cm^-1^; ^1^H and ^13^C NMR (850 and 212.5 MHz, respectively) (Table S1); negative HR-ESIMS *m/z* 547.1093 [M - H]^-^ (calculated for C_25_H_23_O_14_, 547.1088).

**Table S1.** ^1^H (850 MHz) and ^13^C NMR (212.5 MHz) data for compounds **M-1** and **M-2** in CD_3_OD (δ ppm).^a^

| Position | **M-1** |  | **M-2** |  |
| --- | --- | --- | --- | --- |
|  | *δ*_H_ *(J* in Hz) | *δ*_C_ | *δ*_H_ *(J* in Hz) | *δ*_C_ |
| 2 |  | 157.9 |  | 157.6 |
| 3 |  | 133.7 |  | 133.8 |
| 5 |  | 161.6 |  | 161.6 |
| 6 | 6.11 d (2.0) | 98.5 | 6.11 d (2.0) | 98.4 |
| 7 |  | 164.8 |  | 164.8 |
| 8 | 6.31 d (2.0) | 93.3 | 6.31 d (2.0) | 93.2 |
| 9 |  | 157.1 |  | 157.1 |
| 10 |  | 104.1 |  | 104.2 |
| 1' |  | 121.3 |  | 121.3 |
| 2' | 7.94 d (9.0) | 130.8 | 7.93 d (9.0) | 130.8 |
| 3' | 6.77 d (9.0) | 114.6 | 6.78 d (9.0) | 114.5 |
| 4' |  | 160.1 |  | 160.1 |
| 5' | 6.77 d (9.0) | 114.6 | 6.78 d (9.0) | 114.5 |
| 6' | 7.94 d (9.0) | 130.8 | 7.93 d (9.0) | 130.8 |
| 1'' | 5.08 d (7.5) | 102.6 | 5.09 d (7.5) | 102.5 |
| 2'' | 3.34 m | 74.1 | 3.33 m | 74.1 |
| 3'' | 3.31 m | 76.4 | 3.32 m | 76.4 |
| 4'' | 3.18 m | 69.9 | 3.20 m | 69.7 |
| 5'' | 3.27 m | 74.1 | 3.29 m | 73.9 |
| 6''a | 4.10 dd (11.5, 2.0) | 62.9 | 4.17 dd (11.5, 2.0) | 63.3 |
| 6''b | 3.98 dd (11.5, 6.0) |  | 4.02 dd (11.5, 6.0) |  |
| 1''' |  | 172.1 |  | 166.5 |
| 2''' | 2.32 overlap | 28.5 | 3.17 m | 47.7 |
| 3''' | 2.26 overlap | 27.6 |  | 167.1 |
| 4''' |  | 173.0 |  |  |
| OCH_3_ | 3.53 s | 50.7 | 3.53 s | 50.7 |

^a^*J* values are in parentheses and shown in Hz; ^13^C NMR assignments are based on HSQC and HMBC experiments.

*Acid hydrolysis and absolute configuration determination of the sugar moieties of* ***M-1*** *and* ***M-2***

The absolute configuration of the sugar moieties of **M-1** and **M-2** was determined using a modified HPLC-UV-based method.[1, 2] Compounds **M-1** and **M-2** (each 0.3 mg) were hydrolyzed in the presence of 1 N HCl at 80 °C for 2 h, and EtOAc was used for the extraction. The aqueous layer was neutralized by repeated evaporation under vacuum and dissolved in anhydrous pyridine (0.5 mL) with the addition of L-cysteine methyl ester hydrochloride (1.0 mg). After the reaction mixture was heated at 60 °C for 1 h, *O*-tolyl isothiocyanate (50 μL) was added, and the mixture was maintained at 60 °C for 1 h. The reaction product was evaporated using a vacuum evaporator and dissolved in methanol. Next, the dissolved reaction product was diretly analyzed by LC/MS [100% MeOH → 80% MeOH/H_2_O (0**–**30 min) gradient solvent system, 100% MeOH (31–41 min), 0% MeOH (42–52 min), and a flow rate of 0.3 mL/min] using an analytical Kinetex C_18_ 100 Å column (100 mm × 2.1 mm i.d., 5 μm). The sugar moieties in the compounds (**M-1** and **M-2**) were identified as D-glucose based on a comparison with the retention time of an authentic sample (D-glucose *t*_R_ = 19.3 min).

*SARS-CoV-2 Plaque Formation Assay*

Live SARS-CoV-2 tests were conducted in Biosafety Level 3 (BSL3) approved facilities by personnel who had received training in handling BSL3 infectious agents while adhering to standardized safety and decontamination procedures. Calu-3 cells were plated at 5 × 105 cells/well in 6-well plates in Dulbecco’s-Modified Eagle Medium (DMEM, Thermo Fisher Scientific, Waltham, MA, USA) supplemented with 10% FBS (Thermo Fisher Scientific, Waltham, MA, USA). Twenty-four hours later, cells were treated with **K-4** or **M-4** for 4 hours before to infection, and then incubated with a virus suspension at a multiplicity of infection (MOI) of 0.001 for 1 hour of SARS-CoV-2 Omicron. Supernatants were removed after 1 hour of incubation at 37°C and each well received 3 mL of 1% agarose overlay (Sigma Aldrich, St. Louis, MO, USA) dissolved in complete medium. Alternatively, Calu-3 cells were cotreated for 1 hour with chemicals and a viral solution comprising 0.001 MOI in a total volume of 500 μL complete media. The agarose overlay was added as previously mentioned after the supernatants were discarded. Cells were fixed using 4% formaldehyde/PBS solution for 10 minutes after 3 days, and then stained for 1 hour with 0.1% crystal violet (Sigma Aldrich, St. Louis, MO, USA) in 70% methanol. The plaques were counted under a microscope and photographed using the EVOS M5000 Imaging System (Thermo Fisher Scientific Inc., Waltham, MA, USA).

*Transcriptomic Analysis*

Raw sequence reads produced by the sequencer were cleaned using Trim Galore version 0.6.5. The cleaned reads were aligned on human reference genome (GRCh38.p13) and quantified using Subread version 2.0.3 [3]. Differentially expressed genes (DEGs) were identified using DESeq2 version 1.36.0 [4] with the P-value < 0.05 and | Log2(Fold change) | > 2 threshold for significance. Gene ontology (GO), disease ontology (DO), and Kyoto Encyclopedia of Genes and Genomes (KEGG) pathway enriched on significant DEGs were accessed using clusterProfiler version 4.4.4 [5]. The KEGG pathway map colored by fold change of DEGs were generated using Pathview version 1.36.1 [6]. Enrichment scores of each sample on gene sets and cell markers were calculated using GSVA version 1.44.5 [7]. Volcano plots, bar plots, box plots, dot plots and heatmaps were generated using ggplot2 (version 3.4.0) and ComplexHeatmap (version 2.13.1). All statistical analysis and visualizations were performed under R (version 4.2.2) and R studio (Build 576) envelopment.

***Natural Products Library Selection and Structure Characterization***

We initiated our study to discover potential flavonoids as anti-SARS-CoV-2 natural products from our collection of dozens of plants and microorganism-derived extracts assembled through continuous research projects investigating bioactive natural products from diverse natural sources. The LC/MS-based analyses of our extract library, including Global Natural Product Social Molecular Network (GNPS) analysis in combination with our in-house UV library database, revealed that the MeOH extract of black ginger, *Kaempferia parviflora* rhizomes, and the EtOH extract of mulberry (*Morus alba*) fruit were promising samples rich in flavonoids. *K. parviflora*, also known as Thai ginseng, is a medicinal plant belonging to the family Zingiberaceae and is native to northern and northeastern Thailand, where its rhizomes are popular as a commercial health-promoting herb. It has been used in traditional medicine to treat various diseases, including ulcers, gout, allergy, abscesses, and osteoarthritis.[8, 9] The rhizome of *K. parviflora* reportedly has valuable pharmacological effects, including anticancer, antimicrobial, anti-inflammatory, antiallergic, antioxidative, aphrodisiac, neuroprotective, vascular relaxation, and cardioprotective activities,[10] which has allowed thorough investigation of the bioactive phytochemicals of *K. parviflora*[11–13]; consequently, methoxyflavones have been identified as the bioactive components.[11–13] Mulberry (*M. alba*) is famous worldwide for its berries and is an essential food for silkworms (*Bombyx mori* L.). It has been widely cultivated in many countries, especially in Asia. Its fruit has a long history of use as an edible fruit and in traditional medicine.[14] The fruits of mulberry are also internationally used in pies, tarts, wines, jams, smoothies, and tisanes. Several previous phytochemical studies on mulberry fruits have identified bioactive secondary metabolites of *M. alba* fruits, including flavonoids, anthocyanins, and triterpenoids.[15–19]

The MeOH extract of *K. parviflora* rhizomes was sequentially subjected to solvent partitioning with four organic solvents, namely hexane, dichloromethane, ethyl acetate, and *n*-butanol, yielding four fractions. The LC-MS-based analysis of each fraction was conducted using an in-house UV library database, which verified that the hexane-soluble fraction was rich in flavonoids. Phytochemical investigation of the hexane-soluble fraction using repeated column chromatography and semi-preparative high-performance liquid chromatography (HPLC) separation led to the isolation of 12 methoxyflavones (**K-1 – K-12**) (Figure 1). The isolated methoxyflavones were structurally characterized as 5-hydroxy-7-methoxyflavone (**K-1**),[20] 3,7-dimethoxy-5-hydroxyflavone (**K-2**),[21] 5,7,4′-trimethoxyflavone (**K-3**),[20] 5-hydroxy-3,7,3′,4′-tetramethoxyflavone (**K-4**),[22] *trans*-3-hydroxy-5,7-dimethoxyflavanone (**K-5**),[23] 5,7-dimethoxyflavanone (**K-6**),[24] 7,4′-dimethylapigenin (**K-7**),[20] 5,7-dimethoxyflavone (**K-8**),[20] 3,5,7,4′-tetramethoxyflavone (**K-9**),[25] (-)-pinostrobin (**K-10**),[21] 3,7,4′-trimethylkaempferol (**K-11**),[26] and 3,5,7-trimethoxyflavone (**K-12**)[27] by comparing their NMR spectroscopic data with data from previously reported studies and MS data obtained from LC-MS analyses.

The EtOH extract of *M. alba* fruit was subjected to solvent partitioning with four organic solvents (hexane, dichloromethane, ethyl acetate, and *n*-butanol), which yielded four key fractions. LC-MS-based analysis of each fraction, combined with data present in the in-house UV library database, revealed that the EtOAc-soluble fraction was rich in flavonoids. Detailed chemical analysis of the EtOAc-soluble fraction using a combination of column chromatography and semi-preparative HPLC led to the isolation of seven flavonoid glycosides (**M-1 – M-7**), including two new compounds, **M-1** and **M-2** (Figure 1). Compound **M-1** was isolated as a yellowish, amorphous powder. Its molecular formula was determined to be C_26_H_26_O_14_ based on the NMR data (Table S1) and HR-ESIMS data, and it showed a molecular ion peak at *m/z* 561.1248 [M-H]^-^ (calculated for C_26_H_25_O_14_, 561.1244) in the negative-ion mode. The IR spectrum of 1 displayed distinctive absorption bands for the hydroxy (3420 cm^−1^) and *α,β*-unsaturated ketone (1658 cm^−1^) functional units. The ^1^H NMR data (Table S1) of **M-1** combined with the data from the HSQC experiment showed the presence of signals for six aromatic methines at *δ*_H_ 6.11 (1H, d, *J* = 2.0 Hz, H-6)/*δ*_C_ 98.5 (C-6), 6.31 (1H, d, *J* = 2.0 Hz, H-8)/*δ*_C_ 93.3 (C-8), *δ*_H_ 6.77 (2H, d, *J* = 9.0 Hz, H-3′ and H-5′)/*δ*_C_ 114.6 (C-3′ and C-5′), and *δ*_H_ 7.94 (2H, d, *J* = 9.0 Hz, H-2′ and H-6′)/*δ*_C_ 130.8 (C-2′ and C-6′), which indicated a typical kaempferol skeleton.[28] Furthermore, the presence of signals for a glucose moiety, including an anomeric proton/carbon at *δ*_H_ 5.08 (1H, d, *J* = 7.5 Hz, H-1′′) and *δ*_C_ 102.6 (C-1′′), a succinyl group at *δ*_H_ 2.32 (2H, overlap, H-2′′′)/*δ*_C_ 28.5 (C-2′′′) and *δ*_H_ 2.26 (2H, overlap, H-3′′′)/*δ*_C_ 27.6 (C-3′′′), as well as one methoxy group at *δ*_H_ 3.53(3H, s)/*δ*_C_ 50.7 was observed. Thorough scrutiny of the NMR spectral data suggested that the structure of **M-1** was very similar to that of **M-3**, as identified in the present study[29]; however, the apparent difference between the structures of **M-1** and **M-3** was identified in the B-ring due to the discrepancy in the NMR signals corresponding to the B-ring and additional methoxy groups. The kaempferol skeleton and linkage positions of the glucose and succinyl units were unambiguously confirmed by interpretation of the key HMBC correlations and COSY correlations, as shown in Figure S1. Notably, the methoxy group at C-4′′′ was determined using the key HMBC correlation of OCH_3_/C-4′′′ (Figure S1). In conclusion, the structure of **M-1** was determined to be kaempferol-3-*O*-glucoside 6 ‘’-succinate methyl ester. Compound **M-2**, obtained as a yellowish amorphous powder, possessed the molecular formula C_25_H_24_O_14_ as determined by the negative-ion mode HR-ESI-MS data at *m*/*z* 547.1093 [M-H]^-^ (calculated for C_25_H_23_O_14_, 547.1088) and NMR data (Table S1). The ^1^H and ^13^C NMR spectra of **M-2** (Table S1), combined with HSQC and HMBC experiments, closely resembled those of **M-1**. The only minor difference in their chemical shifts was at the level of the side chain; the succinate methyl ester in compound **M-1** was replaced by the malonate methyl ester in compound **M-2**. The complete structure of **M-2** was further determined using 2D NMR analysis (^1^H-^1^H COSY and HMBC) (Figure S1). Accordingly, **M-2** was characterized as kaempferol-3-*O*-glucoside 6"-malonate methyl ester. Finally, the absolute configuration of the glucose moiety in **M-1** and **M-2** was determined by acid hydrolysis, which resulted in the production of glucopyranose. The *β*-D-glucopyranose was determined by comparing the retention time (*t*_R_ 19.3 min) of its thiocarbamoyl-thiazolidine derivative with that of the standard sample of D-glucopyranose by LC-MS analysis and the coupling constant (*J* = 7.5 Hz) of the anomeric proton signal, indicative of the *β*-form for glucose.[30] The other isolated flavonoid glycosides were identified as 6’-succinyl quercetin-3-glucoside (**M-3**),[29] rutin (**M-4**),[31] isoquercetin (**M-5**),[31] quercetin-3-glucoside 6"-malonate methyl ester (**M-6**),[32] and kaempferol 3-rutinoside (**M-7**)[28, 33] by comparing their respective NMR data with previously reported data, in addition to LC-MS analysis.

**Figure S1.** ^1^H−^1^H COSY ( ) and key HMBC () correlations of compounds **M-1** and **M-2**.

**Figure S2.** Modeling SARS-CoV-2 infection using hiPSC differentiation into lung organoids (LOs) (n = 3). (A) Schematic of protocol and timeline. (B) The hiPSCs were positive for pluripotency markers, such as OCT4 and NANOG. (C) Phase-contrast image of lung organoids (LOs) at 36 days. (D) The expression of lung cell markers of lung organoid differentiation was estimated by real-time PCR. (E) Immunofluorescent staining results detecting lung cell-specific markers, including EpCAM, NKX2.1, SOX2, and SOX9. Scale bar: 20 μm. (F) Control (GFP) or SARS-CoV-2 Omicron spike-GFP PV infection in lung organoid models. Values indicate means ± SEM. (n = 3, *** p < 0.001, **** < 0.0001 vs. control group).


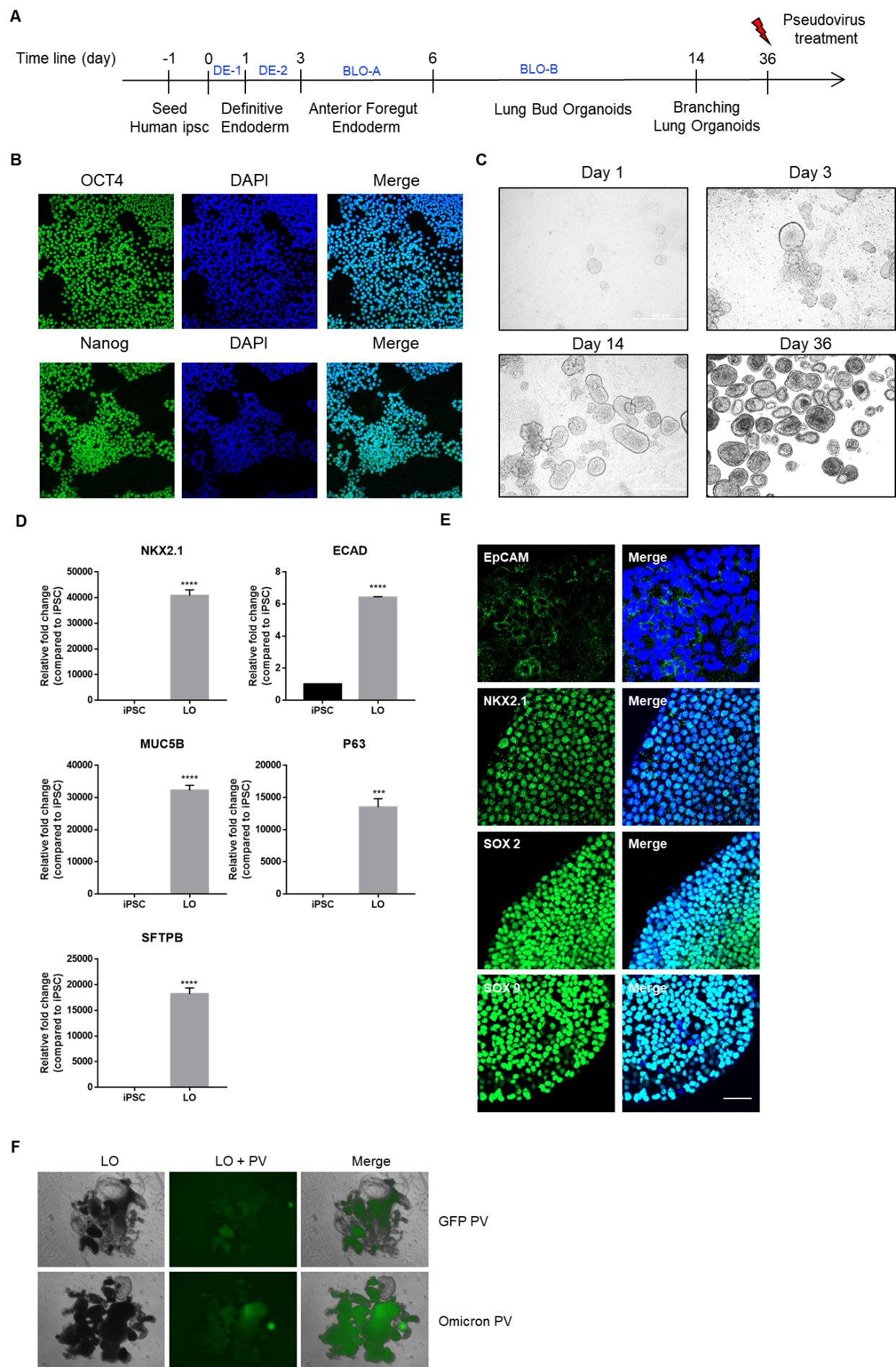


**Figure S3**. Inhibition of SARS-CoV-2 variant infection of Calu-3 cells. (A, B) The number of plaques was significantly different in Calu-3 cells treated with **K-4** or **M-4** compared to DMSO before infection of SARS-CoV-2 Omicron. Infectivity was tested using compounds of **K-4** and **M-4** (10 µM) against the SARS-CoV-2 Delta (C, D) or Omicron (E, F) variant. Values indicate means ± SEM. (n = 3, * p < 0.05, ** < 0.01 vs. control group).


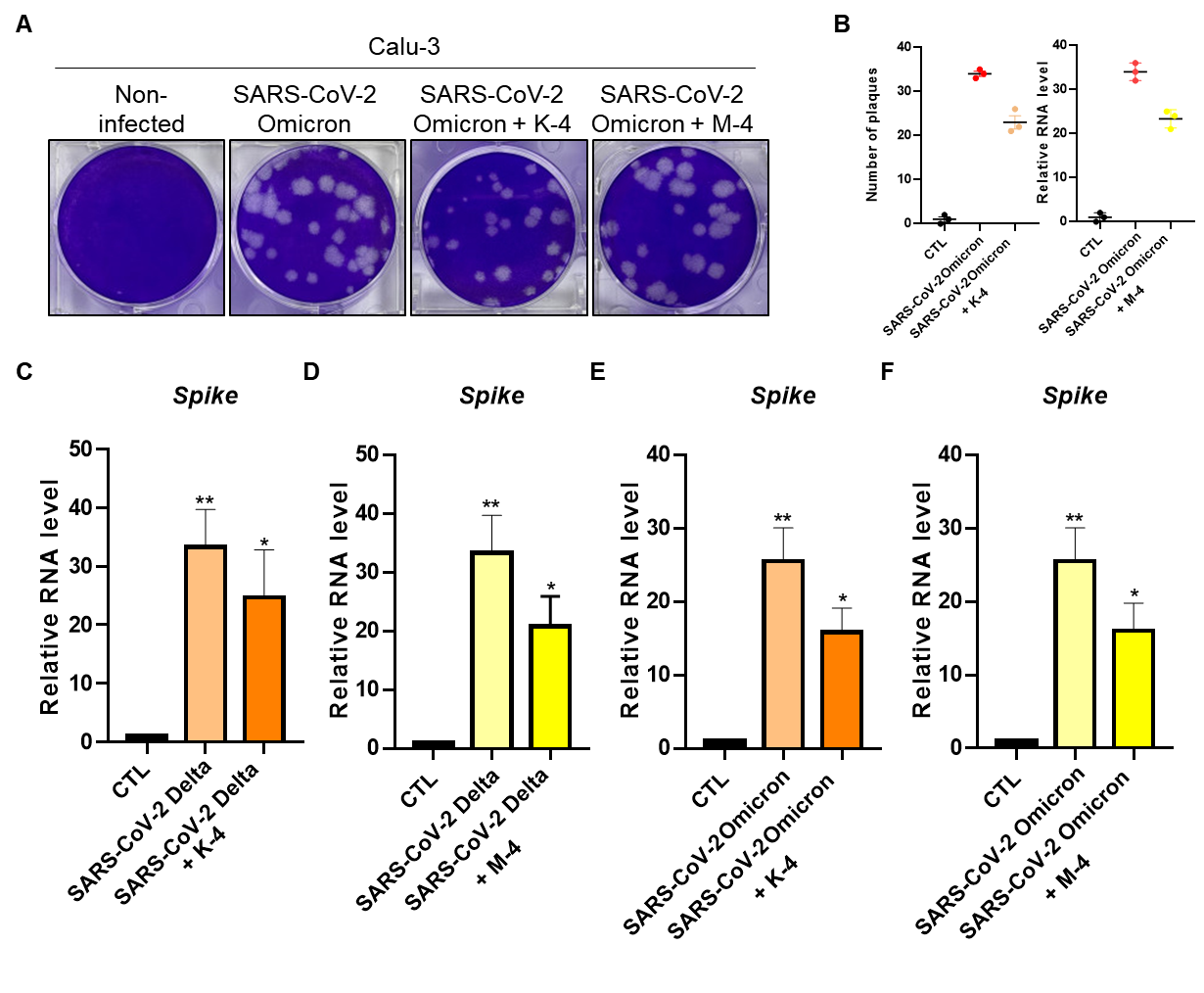


**Figure S4**. The enriched gene sets in control (GFP) or pseudovirus of SARS-CoV-2 Omicron variant infected in human LOs (n = 3). (A, B) The enriched gene sets of up- or downregulated genes are associated with the SARS-CoV-2-related gene set.


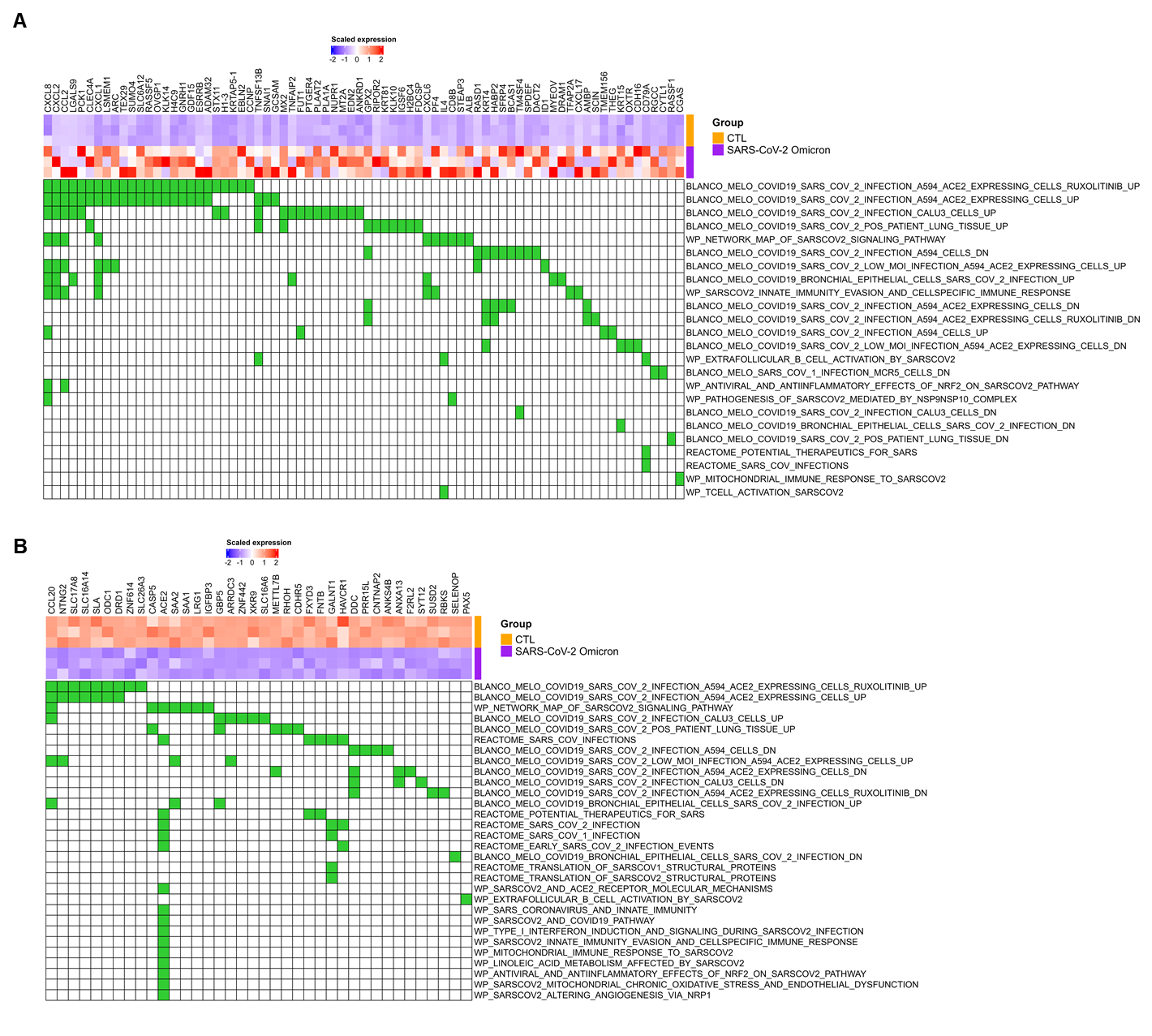


**Figure S5**. Differentially expressed gene (DEG) and gene ontology (GO) analyses of transcriptome in the control (GFP) and SARS-CoV-2 Omicron variant pseudovirus-infected human LOs (n = 3). (A) Genes of significantly up- or downregulated DEGs. (B) The top 20 GO terms associated with increased or decreased DEG in LOs. (C) Gene set variation analysis (GSVA) results.


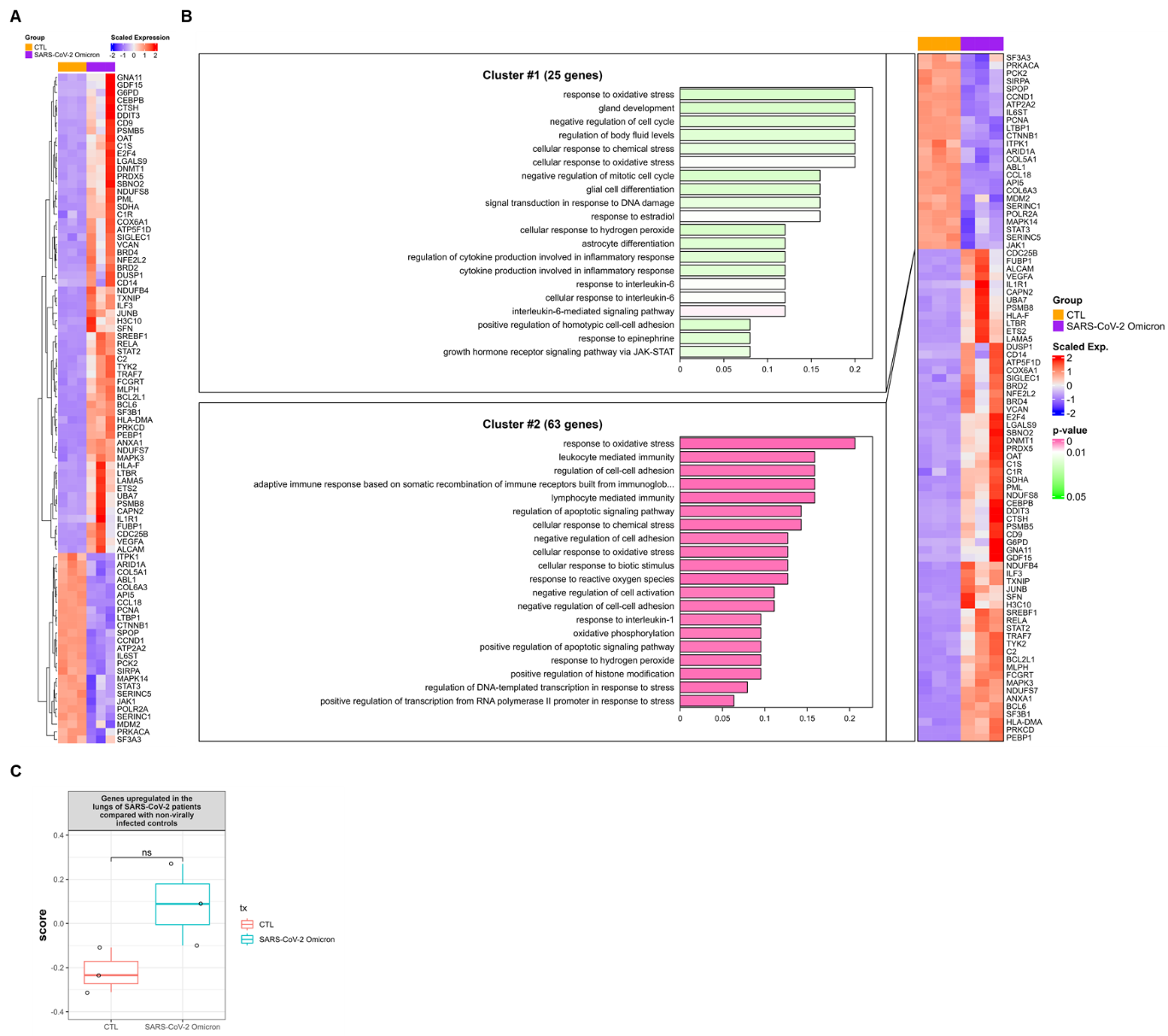


**Figure S6**. The subtype variation analysis. Gene set variation analysis (GSVA) of PV-uninfected or infected LOs on gene sets and cell markers (n = 3).


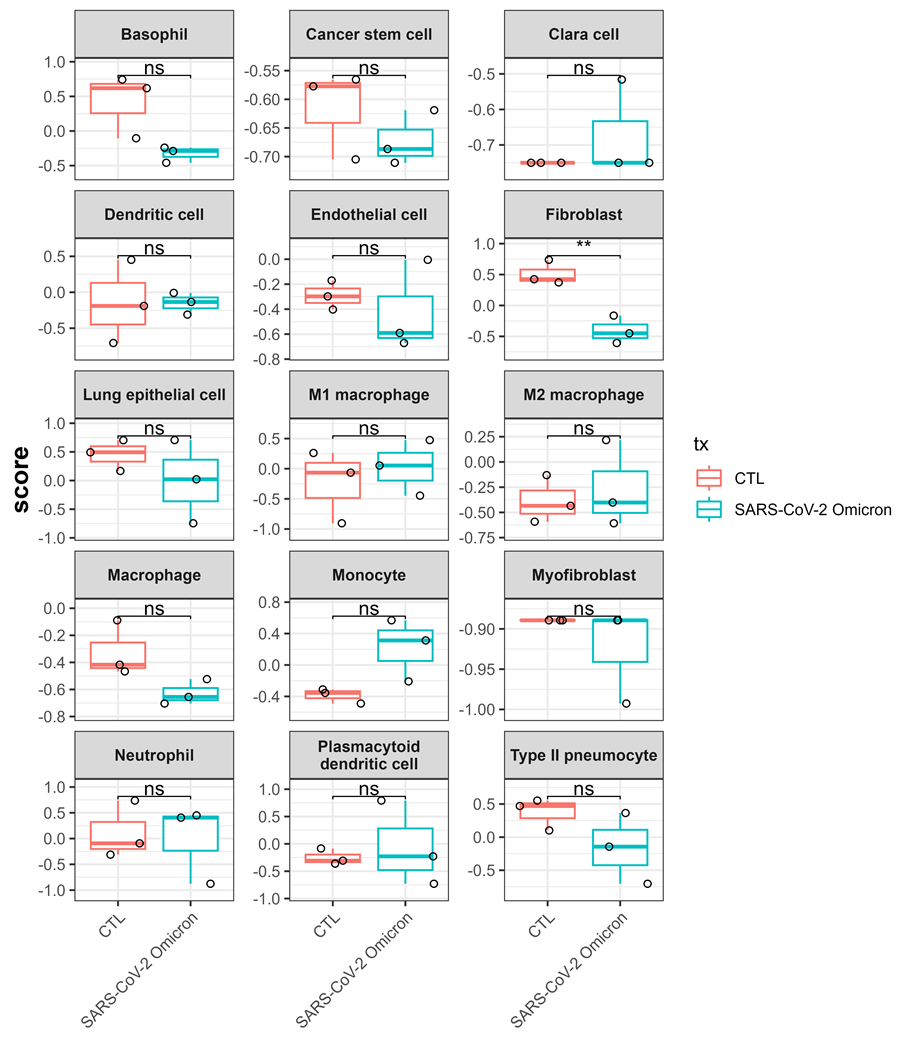


**Figure S7**. Differentially expressed gene (DEG), gene ontology (GO), and gene set variation analysis (GSVA) of PV-uninfected or infected LOs on gene sets of the COVID-19 patient samples (n = 3). (A) Heatmap of enrichment by referring to the COVID-19 patient samples database. (B) The top-rank GO terms are associated with increased or decreased DEG in LOs. (C) Gene set variation analysis (GSVA) results.


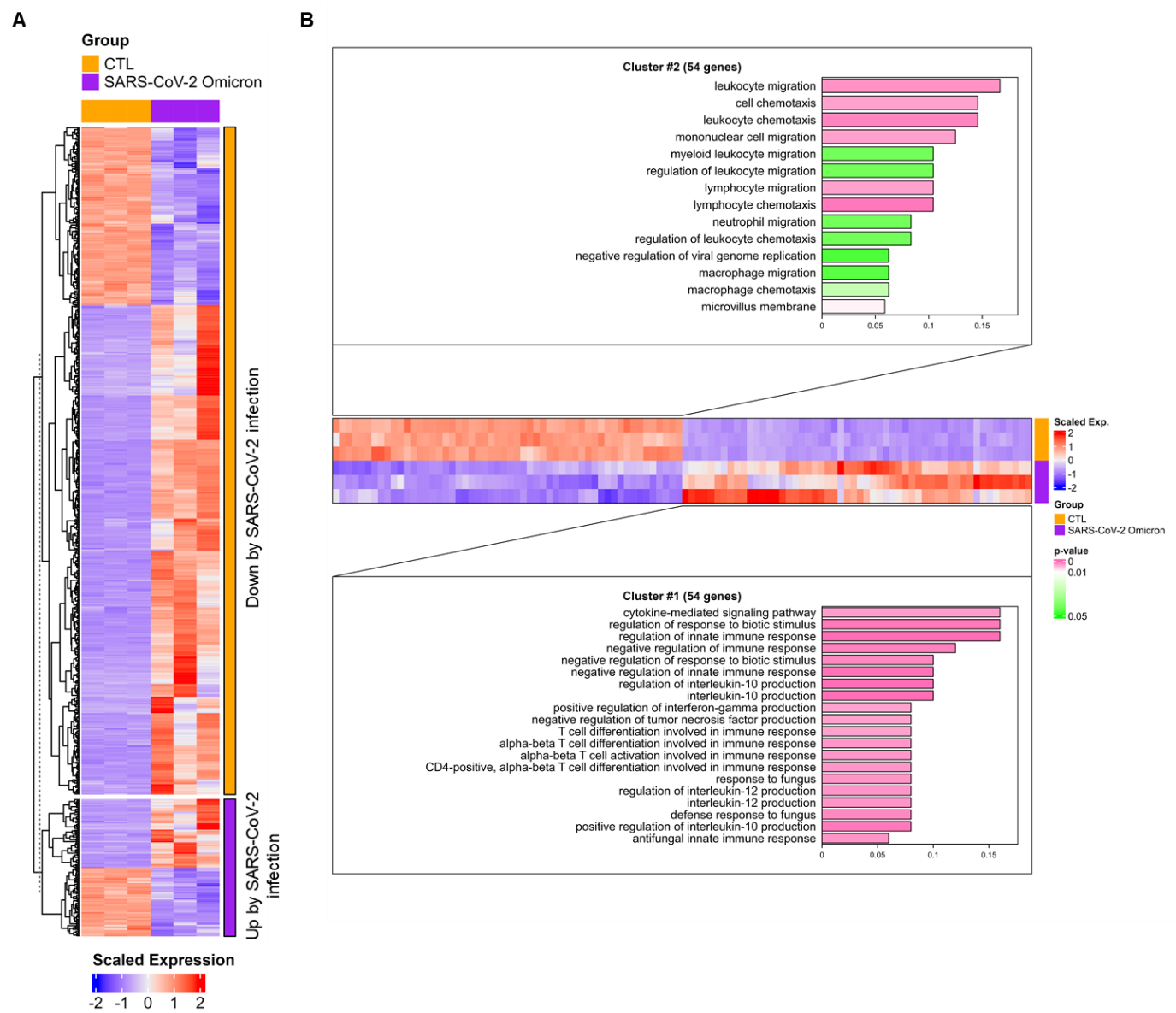


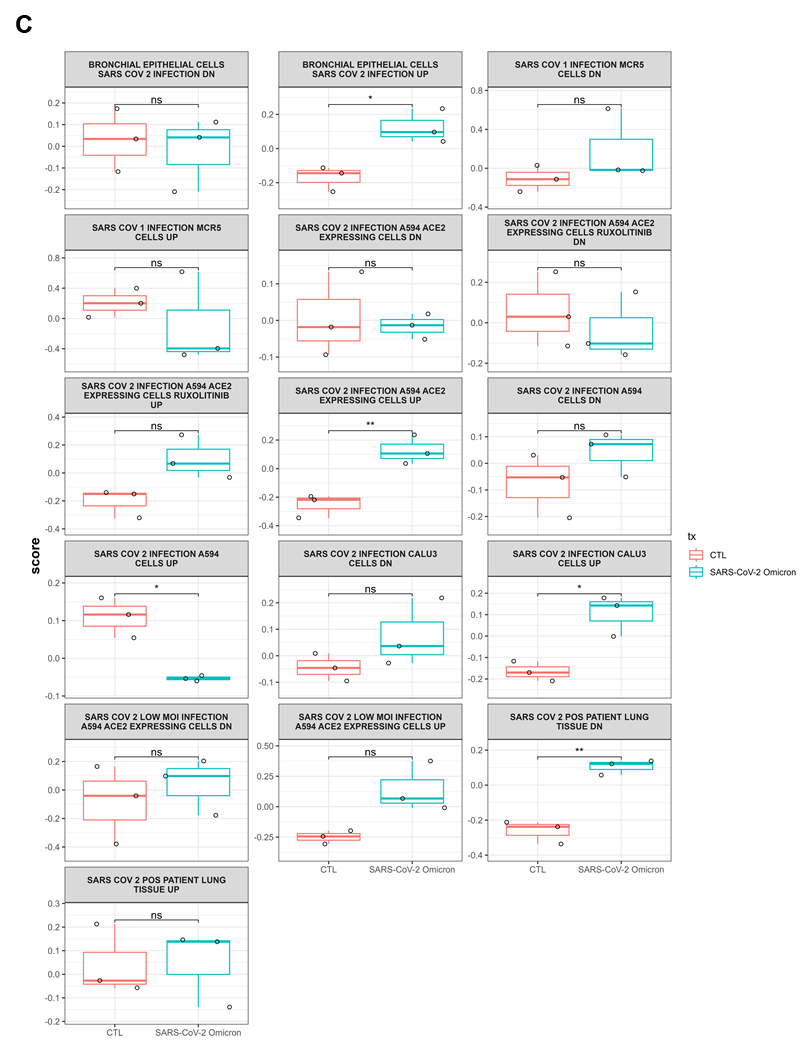


**Figure S8**. Kyoto Encyclopedia of Genes and Genomes (KEGG) pathway and differentially expressed genes (DEGs) (n = 3). (A) KEGG pathway of ‘Coronavirus disease - COVID-19’. (B) Heatmap for the enrichment analysis of DEGs.

**Figure S9**. Gene ontology (GO) and differentially expressed gene (DEG) of PV-uninfected or infected LOs (n = 3). (A) Pathway-related GO analysis. (B) Heatmap for the enrichment analysis of DEGs.


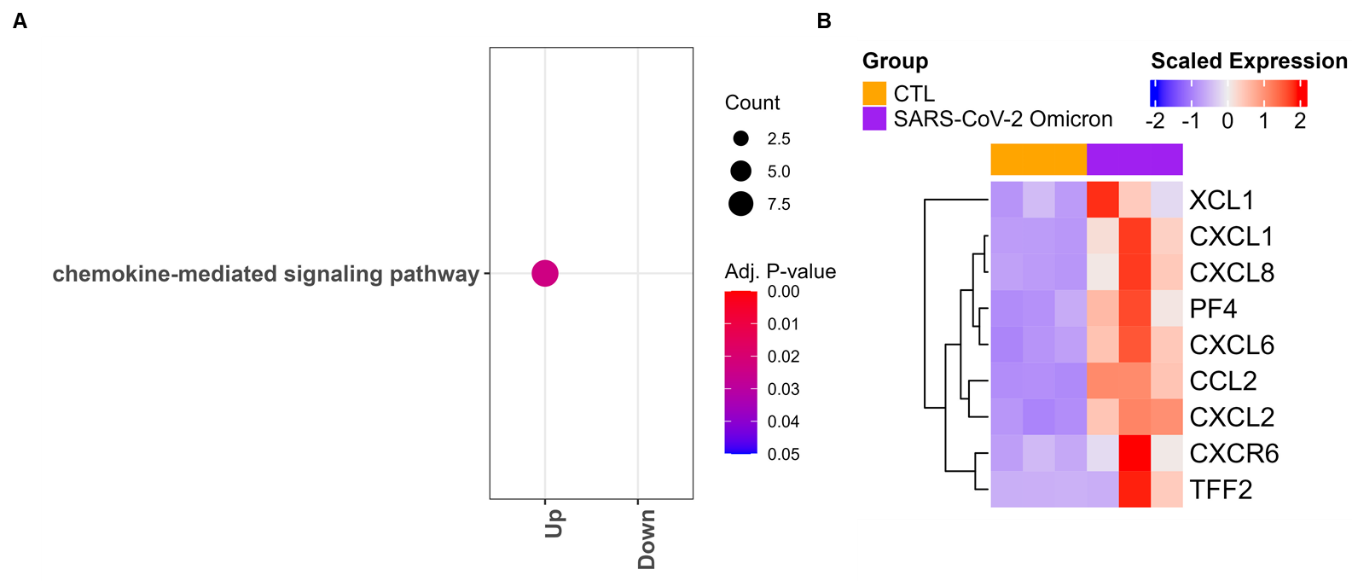


**Figure S10**. Disease Ontology (DO) enrichment analyses. The top 8 terms in the DO enrichment analysis (n = 3). The x-axis shows the number of genes ratio in each term. The p-value of each term is indicated by color according to the legend.


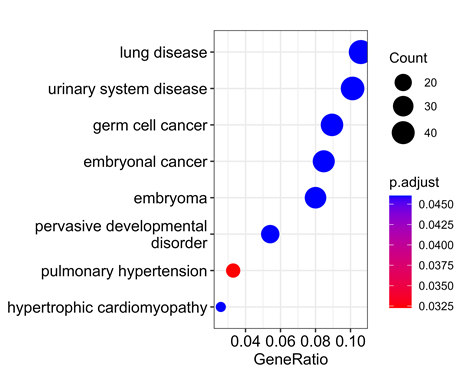


***HR-MS, and 1D- and 2D-NMR data for compounds M-1 and M-2***

**Figure S11.** HR-ESI-MS of compound **M-1**.


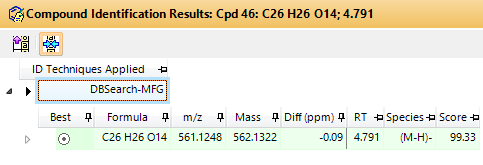


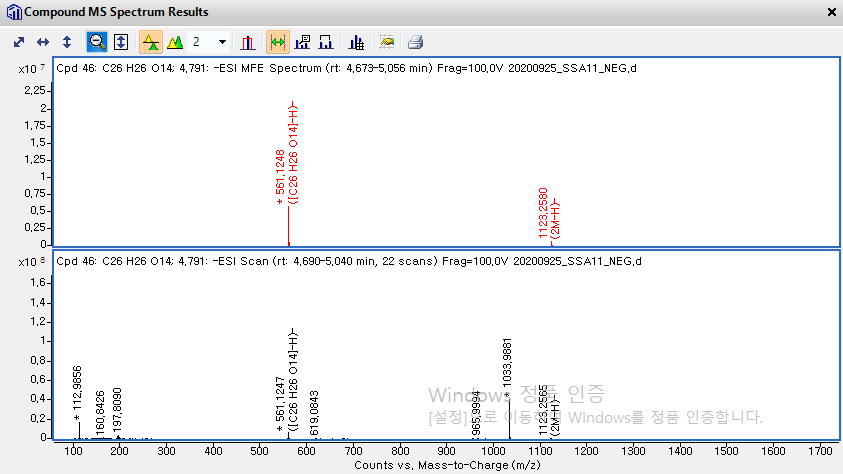


**Figure S12.** ^1^H NMR spectrum of compound **M-1**.

**Figure S13.** HSQC spectrum of compound **M-1**.

**Figure S14.** ^1^H−^1^H COSY spectrum of compound **M-1**.

**Figure S15.** HMBC spectrum of compound **M-1**.


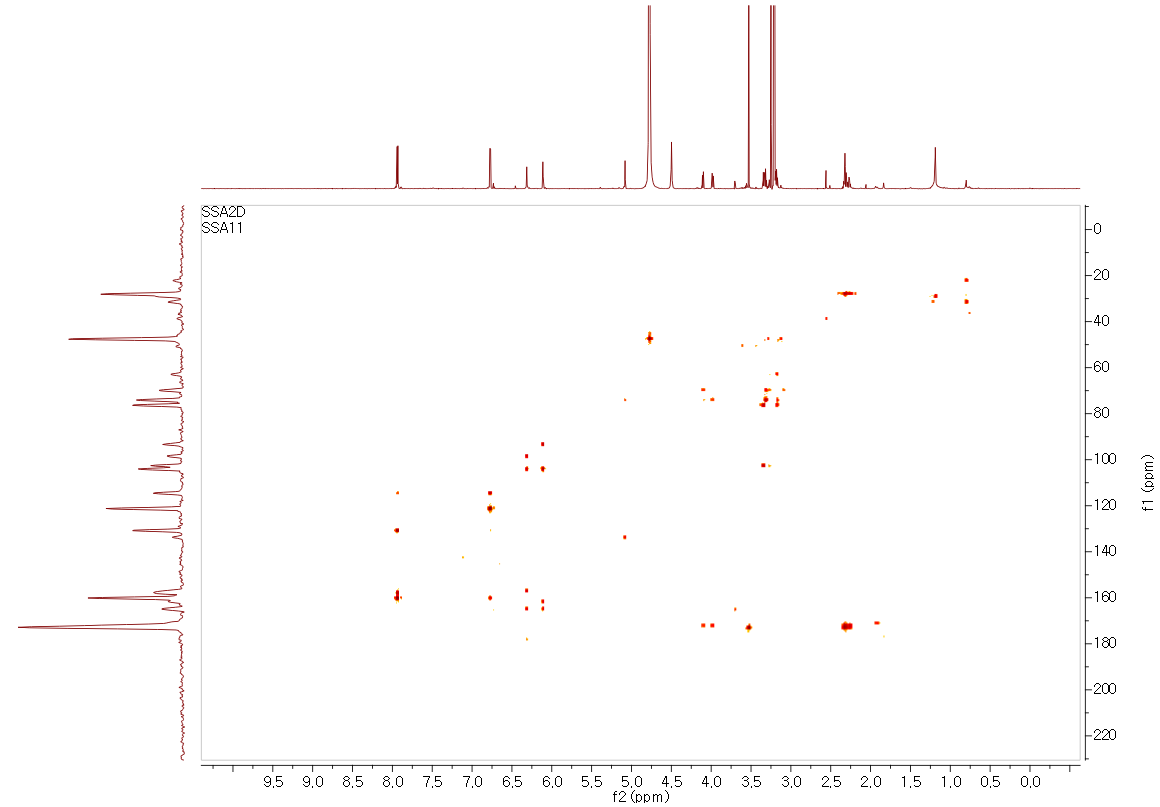


**Figure S16.** NOESY spectrum of compound **M-1**.

**Figure S17.** Retention time of reaction product of thiocarbamoyl-thiazolidine derivative of glucose (A) from compound **M-1** and β-ᴅ-glucose standard (B).

**
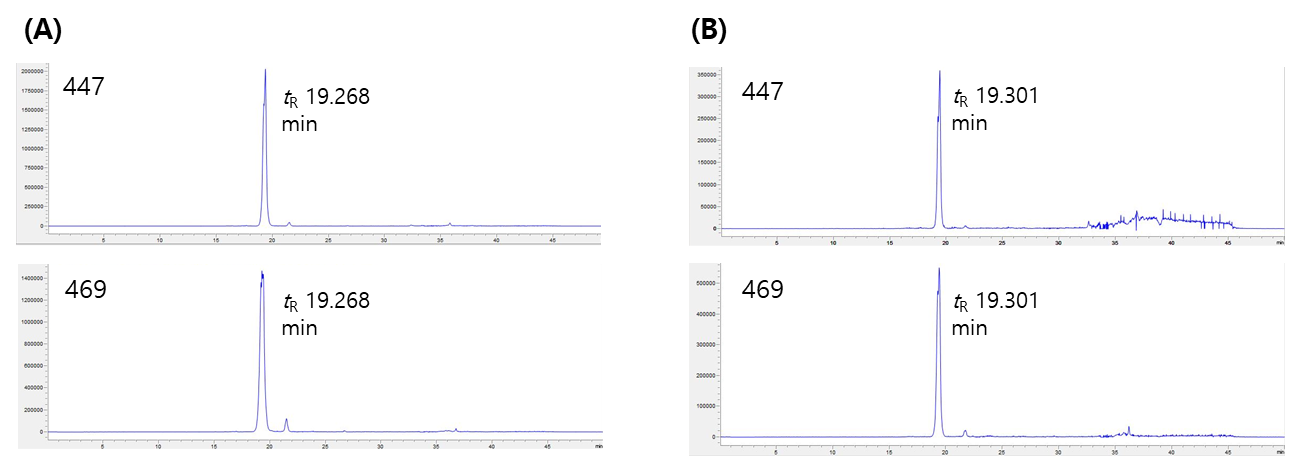
**

**Figure S18.** HR-ESI-MS of compound **M-2**.


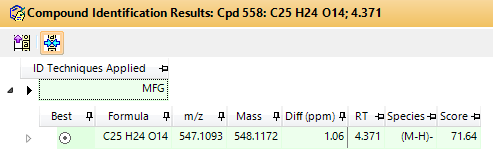


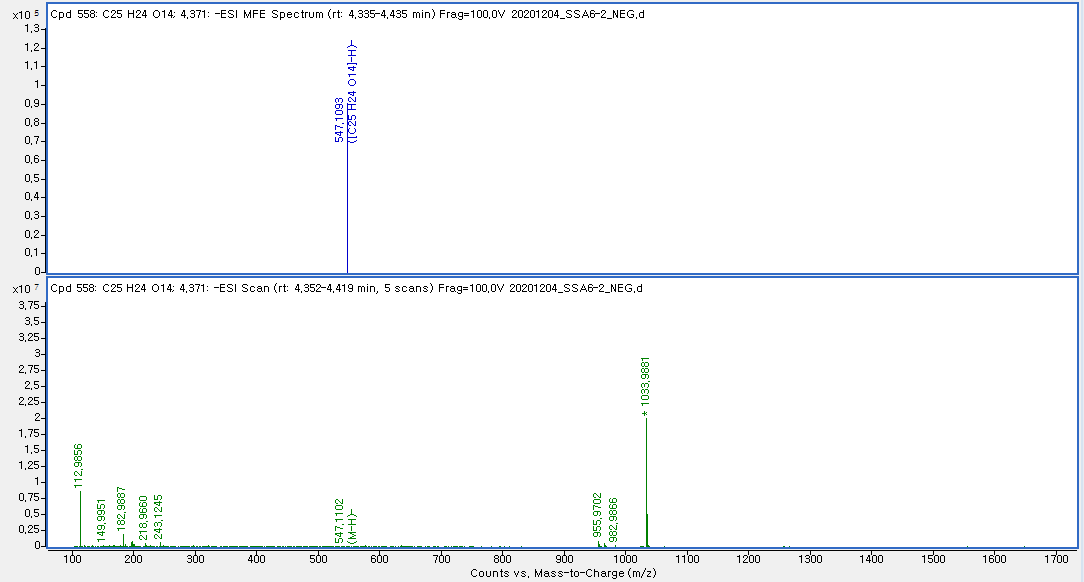


**Figure S19.** ^1^H NMR spectrum of compound **M-2**.

**Figure S20.** HSQC spectrum of compound **M-2**.

**Figure S21.** ^1^H−^1^H COSY spectrum of compound **M-2**.

**Figure S22.** HMBC spectrum of compound **M-2**.

**Figure S23.** NOESY spectrum of compound **M-2**.

# Supplemental References

1. Jo MS, Lee S, Yu JS, Baek SC, Cho YC, Kim KH. Megastigmane Derivatives from the Cladodes of Opuntia humifusa and Their Nitric Oxide Inhibitory Activities in Macrophages. J Nat Prod. 2020;83:684–92.

2. Tanaka T, Nakashima T, Ueda T, Tomii K, Kouno I. Facile discrimination of aldose enantiomers by reversed-phase HPLC. Chem Pharm Bull (Tokyo). 2007;55:899–901.

3. Liao Y, Smyth GK, Shi W. The Subread aligner: fast, accurate and scalable read mapping by seed-and-vote. Nucleic Acids Res. 2013;41.

4. Love MI, Huber W, Anders S. Moderated estimation of fold change and dispersion for RNA-seq data with DESeq2. Genome Biol. 2014;15:1–21.

5. Wu T, Hu E, Xu S, Chen M, Guo P, Dai Z, et al. clusterProfiler 4.0: A universal enrichment tool for interpreting omics data. Innov (Cambridge. 2021;2.

6. Luo W, Brouwer C. Pathview: an R/Bioconductor package for pathway-based data integration and visualization. Bioinformatics. 2013;29:1830–1.

7. Hänzelmann S, Castelo R, Guinney J. GSVA: Gene set variation analysis for microarray and RNA-Seq data. BMC Bioinformatics. 2013;14:1–15.

8. Saokaew S, Wilairat P, Raktanyakan P, Dilokthornsakul P, Dhippayom T, Kongkaew C, et al. Clinical Effects of Krachaidum ( Kaempferia parviflora): A Systematic Review. J Evid Based Complementary Altern Med. 2017;22:413–28.

9. Toda K, Hitoe S, Takeda S, Shimoda H. Black ginger extract increases physical fitness performance and muscular endurance by improving inflammation and energy metabolism. Heliyon. 2016;2.

10. Chen D, Li H, Li W, Feng S, Deng D. Kaempferia parviflora and Its Methoxyflavones: Chemistry and Biological Activities. Evid Based Complement Alternat Med. 2018;2018.

11. Wu L, Liu H, Li L, Liu H, Yang K, Liu Z, et al. 5,7,3’,4’-Tetramethoxyflavone exhibits chondroprotective activity by targeting β-catenin signaling in vivo and in vitro. Biochem Biophys Res Commun. 2014;452:682–8.

12. Azuma T, Tanaka Y, Kikuzaki H. Phenolic glycosides from Kaempferia parviflora. Phytochemistry. 2008;69:2743–8.

13. Okabe Y, Shimada T, Horikawa T, Kinoshita K, Koyama K, Ichinose K, et al. Suppression of adipocyte hypertrophy by polymethoxyflavonoids isolated from Kaempferia parviflora. Phytomedicine. 2014;21:800–6.

14. Vijayan K, Chauhan S, Das NK, Chakraborti SP, Roy BN. Leaf yield component combining abilities in mulberry (Morus spp.). Euphytica. 1997;98:47–52.

15. Sass-Kiss A, Kiss J, Milotay P, Kerek MM, Toth-Markus M. Differences in anthocyanin and carotenoid content of fruits and vegetables. Food Res Int. 2005;38:1023–9.

16. Zhao S, Park CH, Li X, Kim YB, Yang J, Sung GB, et al. Accumulation of Rutin and Betulinic Acid and Expression of Phenylpropanoid and Triterpenoid Biosynthetic Genes in Mulberry (Morus alba L.). J Agric Food Chem. 2015;63:8622–30.

17. Lee SR, Park JY, Yu JS, Lee SO, Ryu JY, Choi SZ, et al. Odisolane, a Novel Oxolane Derivative, and Antiangiogenic Constituents from the Fruits of Mulberry (Morus alba L.). J Agric Food Chem. 2016;64:3804–9.

18. Oki T, Kobayashi M, Nakamura T, Okuyama A, Masuda M, Shiratsuchi H, et al. Changes in radical-scavenging activity and components of mulberry fruit during maturation. J Food Sci. 2006;71.

19. Jeong JY, Jo YH, Kim SB, Liu Q, Lee JW, Mo EJ, et al. Pancreatic lipase inhibitory constituents from Morus alba leaves and optimization for extraction conditions. Bioorg Med Chem Lett. 2015;25:2269–74.

20. Tran TD, Yeon SC, Kim J, Hyun PK, Kim S, Park H. Synthesis and PGE2 inhibitory activity of 5,7-dihydroxyflavones and their O-methylated flavone analogs. Arch Pharm Res. 2003;26:345–50.

21. Chou TH, Chen JJ, Lee SJ, Chiang MY, Yang CW, Chen IS. Cytotoxic flavonoids from the leaves of Cryptocarya chinensis. J Nat Prod. 2010;73:1470–5.

22. Nguyen TKP, Nguyen KPP, Kamounah FS, Zhang W, Hansen PE. NMR of a series of novel hydroxyflavothiones. Magn Reson Chem. 2009;47:1043–54.

23. Tarbeeva D V., Fedoreev SA, Veselova M V., Kalinovskii AI, Gorovoi PG, Vishchuk OS, et al. Polyphenolic Metabolites from Iris pseudacorus Roots. Chem Nat Compd 2015 513. 2015;51:451–5.

24. Korenaga T, Hayashi K, Akaki Y, Maenishi R, Sakai T. Highly enantioselective and efficient synthesis of flavanones including pinostrobin through the rhodium-catalyzed asymmetric 1,4-addition. Org Lett. 2011;13:2022–5.

25. Chen HJ, Chung CP, Chiang W, Lin YL. Anti-inflammatory effects and chemical study of a flavonoid-enriched fraction from adlay bran. Food Chem. 2011;126:1741–8.

26. Dae SJ, Han AR, Park G, Jhon GJ, Seo EK. Flavonoids and aromatic compounds from the rhizomes of Zingiber zerumbet. Arch Pharm Res. 2004;27:386–9.

27. Sookkongwaree K, Geitmann M, Roengsumran S, Petsom A, Danielson UH. Inhibition of viral proteases by Zingiberaceae extracts and flavones isolated from Kaempferia parviflora. Pharmazie. 2006;61:717–21.

28. Kazuma K, Noda N, Suzuki M. Malonylated flavonol glycosides from the petals of Clitoria ternatea. Phytochemistry. 2003;62:229–37.

29. Kawai Y, Ishisaka A, Saito S, Uchida K, Shibata N, Kobayashi M, et al. Immunochemical detection of flavonoid glycosides: development, specificity, and application of novel monoclonal antibodies. Arch Biochem Biophys. 2008;476:124–32.

30. Baek SC, Yi SA, Lee BS, Yu JS, Kim JC, Pang C, et al. Anti-Adipogenic Polyacetylene Glycosides from the Florets of Safflower ( Carthamus tinctorius). Biomedicines. 2021;9:1–12.

31. Duru CE, Umar HIU, Duru IA, Enenebeaku UE, Ngozi-Olehi LC, Enyoh CE. Blocking the interactions between human ACE2 and coronavirus spike glycoprotein by selected drugs: a computational perspective. Environ Anal Heal Toxicol. 2021;36.

32. Danieli B, Bertario A, Carrea G, Redigolo B, Secundo F, Riva S. Chemo-enzymatic Synthesis of 6″-O-(3-Arylprop-2-enoyl) Derivatives of the Flavonol Glucoside Isoquercitrin. Helv Chim Acta. 1993;76:2981–91.

33. Han JT, Bang MH, Chun OK, Kim DO, Lee CY, Baek NI. Flavonol glycosides from the aerial parts of Aceriphyllum rossii and their antioxidant activities. Arch Pharm Res. 2004;27:390–5.
